# Selective Attention Dynamically Modulates the Hierarchical Order of Perceptual and Conceptual Representations

**DOI:** 10.1002/hbm.70359

**Published:** 2025-09-20

**Authors:** Yu Zhou, Liang Zhang, Nikolai Axmacher, Daniel Pacheco Estefan, Dahui Wang, Yujian Dai, Xiaojing Peng, Shixiang Liu, Gui Xue

**Affiliations:** ^1^ State Key Laboratory of Cognitive Neuroscience and Learning & IDG/McGovern Institute for Brain Research, Beijing Normal University Beijing China; ^2^ Department of Neuropsychology, Institute of Cognitive Neuroscience, Faculty of Psychology Ruhr University Bochum Bochum Germany; ^3^ School of Systems Science Beijing Normal University Beijing China; ^4^ Teachers College of Beijing Union University Beijing China; ^5^ Chinese Institute for Brain Research Beijing People's Republic of China

**Keywords:** feature representation, magnetoencephalography, memory retrieval, selective attention, visual perception

## Abstract

Mounting evidence suggests that information processing in the visual hierarchy involves a progression from low‐level perceptual to high‐level conceptual features during visual perception, and a reverse traversal during memory retrieval. However, the nature of this processing hierarchy and its modulation by selective attention remain unclear. By using the drift‐diffusion model, we found that slower reaction times for conceptual versus perceptual tasks were primarily due to differences in decision boundaries and nondecision times, which were not compensated by faster evidence accumulation for conceptual features. Using single‐trial multivariate decoding of magnetoencephalography (MEG) data, we tracked the temporal dynamics of feature representation during visual perceptual and mnemonic tasks. During perception, selective attention reversed the onset times of perceptual and conceptual features in occipital and parietal lobes, enabling earlier detection of conceptual features. Stronger theta oscillation interactions between occipital and temporal regions during task preparation correlated with earlier onset times of target features in the occipital lobe. During retrieval, selective attention led to earlier peak times for perceptual compared to conceptual features in the frontal lobe. These findings provide novel insights into the dynamic nature of hierarchical processing during perception and memory retrieval, highlighting the critical role of selective attention in modulating information accumulation speed.

## Graphical abstract

Using MEG and multivariate decoding, we show that selective attention alters the temporal dynamics of perceptual and conceptual object features. Attention led to earlier representation of conceptual features during perception and prioritized perceptual reconstruction during memory retrieval, highlighting its role in shaping the temporal flow of feature processing in the brain.

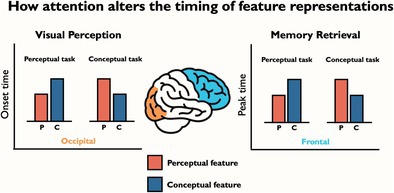

## 1 Introduction

Visual object recognition follows a hierarchical processing pathway in the ventral visual stream, transitioning from low‐level perceptual details to higher‐level conceptual features (Carlson et al. 2013; Cichy et al. 2014; Khaligh‐Razavi et al. 2018; Wang et al. 2022). During object recognition, low‐level perceptual features are generally discriminated more quickly and can be decoded from neural activity earlier than high‐level conceptual features (Clarke et al. 2013; Linde‐Domingo et al. 2019; Mirjalili et al. 2021). In contrast, memory retrieval appears to reverse this sequence: conceptual information is reconstructed more rapidly than perceptual details (Linde‐Domingo et al. 2019; Mirjalili et al. 2021).

Two main perspectives have been proposed to explain these timing differences. One suggested that the faster emergence of certain features simply reflected a fixed sequence of stages in visual processing—lower‐level features were always categorized first, followed by higher‐level features (Brincat and Connor 2004; Serre et al. 2007; Yau et al. 2013). The other posited that relative timing was driven by how much evidence was required for a decision and the rate at which this evidence accumulated (Ratcliff 1978; Ratcliff and McKoon 2008). According to this second view, features that need less evidence (or accumulate evidence more quickly) would be categorized faster.

One potential way to adjudicate between these perspectives is to determine whether selective attention can alter the order in which perceptual and conceptual features emerge during encoding and retrieval. Previous studies suggested that selective attention enhanced target information and suppressed nontarget information (Moore and Zirnsak 2017). For example, studies showed that selective attention could enhance the activation of brain regions responsible for target processing (Maunsell and Treue 2006; Baldauf and Desimone 2014). Beyond boosting activation strength, studies employing multivariate pattern analyses (MVPA) (Kriegeskorte et al. 2006; Hebart and Baker 2018) showed that selective attention could enhance the strength and fidelity of target representations (Long and Kuhl 2018; Grootswagers et al. 2021). Furthermore, attention has been shown to increase the representational dimension (Sheng et al. 2022) and the representational distance among items, allowing for finer discrimination (Nastase et al. 2017) and better memory (Hu et al. 2024).

Building on these findings, we hypothesized that if perceptual and conceptual features are truly constrained by a strictly sequential hierarchy, attention could modulate their latency but not reverse their order. However, if these features are processed in parallel and the processing hierarchy is essentially determined by the different amounts and/or speed of evidence accumulation, attention should be able to prioritize task‐relevant features, for example, leading to earlier detection of conceptual information than perceptual information during visual perception. We also asked whether a similar effect could be observed during memory retrieval, where prior work suggests a reversed processing hierarchy compared to perception (Linde‐Domingo et al. 2019; Mirjalili et al. 2021).

To examine our hypothesis, we systematically selected three features with increasing representational complexity: color, animacy, and real‐world size. Color is a prototypical low‐level perceptual attribute that can be decoded from early visual cortex (Engel 2005; Brouwer and Heeger 2009). Animacy is a mid‐level feature that can be recognized rapidly from coarse perceptual cues but also engages semantic knowledge (Thorat et al. 2019). Real‐world size is a high‐level, abstract concept that typically relies on contextual or semantic inference (Konkle and Caramazza 2013; Khaligh‐Razavi et al. 2018; Wang et al. 2022). We recorded magnetoencephalography (MEG) while participants either (i) categorized objects by a task‐relevant feature (perception task) or (ii) retrieved the task‐relevant feature of a cued object (retrieval task). Using single‐trial multivariate decoding, we quantified the onset and peak latencies of color, animacy, and size representations in occipital, temporal, parietal, and frontal sensors. We found that selective attention reliably accelerated the emergence of target features and resulted in earlier onset times for conceptual features than perceptual features during perception, and reversed peak latencies during memory retrieval. These findings demonstrate that the temporal hierarchy of object representations is not fixed but can be flexibly reordered by task‐driven attention across both perceptual and mnemonic domains.

## 2 Materials and Methods

### 2.1 Participants

Thirty healthy volunteers with normal or corrected‐to‐normal visual acuity and no history of psychiatric or neurological disorders participated in the study. Three of the participants were too drowsy to complete the experiment, so the analysis was conducted on the remaining 27 participants (eight female; mean age = 21.70 ± 2.37 years). All experiments were carried out in accordance with the Declaration of Helsinki. All participants provided written informed consent prior to the experiment, which was approved by the Research Ethics Committee at Peking University and the State Key Laboratory of Cognitive Neuroscience and Learning at Beijing Normal University in China (Approval No. 202009‐29).

### 2.2 Stimuli

**FIGURE 1:**
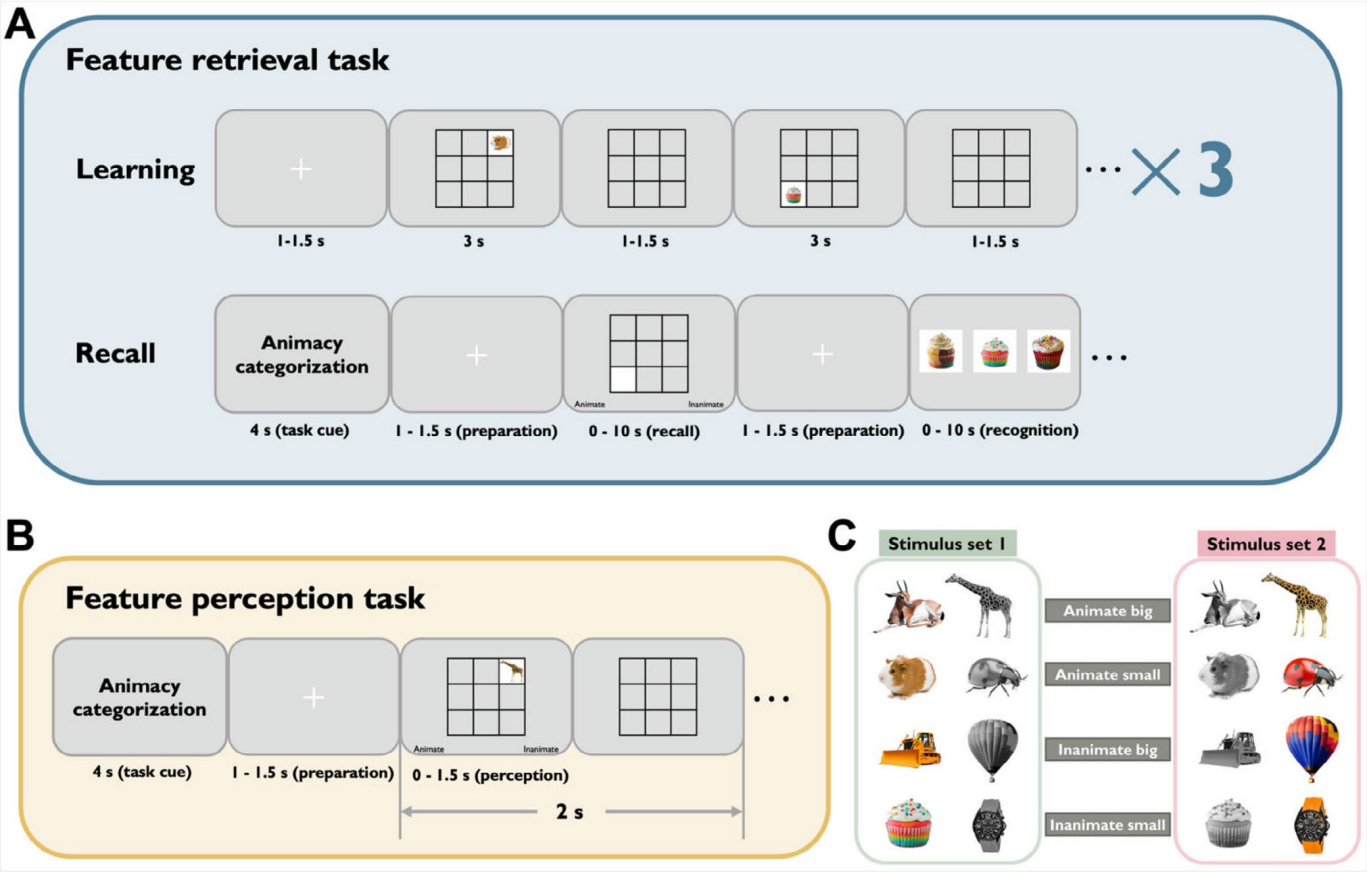
Experimental design and stimuli examples. (A) Feature retrieval task. Participants first learned object‐location associations three times and then recalled the specific features of cued objects, such as color, animacy, or size. The figure illustrates the animacy retrieval task as an example. Participants were asked to retrieve whether the object in the cued location was animate or inanimate. Participants were then tested on their ability to recognize the target objects they had seen during the learning phase. (B) Feature perception task. In this task, participants categorized objects based on the target feature indicated during the task cue period. When presented with the objects, participants were tasked with categorizing them as quickly and accurately as possible. For example, if the task cue was “Animacy categorization,” participants were required to categorize the presented object as either animate or inanimate. (C) Object dimensions. A total of 192 objects varied along three dimensions: One perceptual dimension (colorful or grayscale), and two conceptual dimensions: animacy (animate or inanimate) and real‐world size (small or big). The color of pictures was counterbalanced across two stimulus sets among participants.

To scrutinize the processing hierarchy during both the perception and retrieval stages, it was crucial to independently manipulate the perceptual and conceptual features of the presented objects. In our experiment, objects varied along three orthogonal dimensions: one perceptual dimension (color), where objects were presented either in a colorful or grayscale format, and two conceptual dimensions—animacy and real‐world size. Objects were categorized as either animate or inanimate (animacy dimension) and as either big or small (real‐world size dimension) (Figure 1C). These dimensions were selected to sample low‐, mid‐, and high‐level representations, respectively, and are well established in the literature (Zeki 1983; Brouwer and Heeger 2009; Carlson et al. 2013; Konkle and Caramazza 2013; Cichy et al. 2014; Khaligh‐Razavi et al. 2018; Thorat et al. 2019; Wang et al. 2022).

We utilized 192 distinct images of everyday objects and common animals in the main experiment (plus 24 for practice). The 192 objects were divided into three stimulus sets (Sets 1, 2, and 3), each containing an equal number of animate/inanimate, large/small objects. Half were shown in grayscale and half in color. The three groups were pseudorandomly assigned to each experimental condition and fully counterbalanced across participants. All images were presented at the center of the screen, with a rescaled size of 198 × 198 pixels.

Following existing studies (Schnyer et al. 2007; Brady et al. 2013; He et al. 2020; Zhang et al. 2024), real‐world size was operationalized as a clear, binary distinction: small items could be comfortably held in one hand (e.g., cupcake, wristwatch), whereas large items were big enough to support or seat a person (e.g., chair, hot‐air balloon). To give participants an intuitive anchor, we displayed a cardboard box (≈40 × 18 × 17 cm) before the experiment. This binary scheme, although it loses some granularity, facilitated decoding analyses and enabled direct comparison across all three features.

### 2.3 Procedure

The experiment comprised two parts: the feature retrieval task and the feature perception task. Before each task, participants received verbal instructions and practiced to become familiar with the experimental procedure. The feature retrieval task consisted of eight blocks, each containing three mini‐blocks (24 mini‐blocks in total). Each mini‐block consisted of a learning stage and a recall stage. During the learning stage, a jittered fixation cross (1–1.5 s from a uniform distribution) was followed by a series of objects presented in pseudorandom order within the eight cells surrounding the central cell (no object was shown in the center cell). Each object was displayed for 3 s, with an interstimulus interval jittered around 1–1.5 s from a uniform distribution. Participants were instructed to learn the object‐location associations in preparation for a memory test, with no responses required during the learning stage. The learning process in each mini‐block was repeated three times consecutively. Objects of different types occurred with equal probability across all cells during the entire feature retrieval task.

Following the learning stage, participants engaged in the feature retrieval and object recognition tasks, where they were prompted to recall the features of the cued objects and distinguish target items from lures (Figure 1A). At the beginning of the retrieval stage, a task cue (indicating the color, animacy, or size task) was displayed for 4 s, instructing participants on which feature of the cued object they needed to retrieve (the target feature). A centrally displayed fixation cross appeared for 1–1.5 s, serving as the task preparation stage before the beginning of each retrieval trial. Subsequently, a white square, identical in size to the pictures presented during the learning stage, appeared in any one of the eight cells. Participants were required to retrieve the target feature of the cued object and press the button to provide their answers upon successful recall. For example, in the animacy task, participants had to categorize whether the object in the corresponding location was animate or inanimate. Following this, three pictures were presented on the screen: a target picture shown during the learning stage and two lure pictures resembling the target picture. Participants were tasked with choosing the target picture by pressing the corresponding button. Lure pictures ensured that participants remembered the visual details of the pictures and that the mental process of feature retrieval was based on the retrieved images. Each feature retrieval and object recognition trial allowed participants up to 10 s to respond. Rest breaks were provided after each block, and the feature retrieval task lasted approximately 1 h.

Following the feature retrieval task, participants took a break before commencing the feature perception task, which also utilized a block design (Figure 1B). Each block started with a task cue displayed for 4 s, directing participants on which feature to categorize (the target feature). During each trial, a jittered fixation cross was displayed centrally for 1–1.5 s during the task preparation stage. After this, a random picture appeared in one of the eight cells. Participants were required to rapidly and accurately categorize the object based on the target feature, pressing a button to provide their answers within 1.5 s of seeing the picture. Once the key response was made, the picture vanished from the screen, leaving a blank nine‐cell grid. If the key response was not submitted within 1.5 s of the picture onset, the picture would also disappear. The combined duration of the picture and the blank nine‐cell grid was 2 s. Objects of different types occurred with equal probability in each cell across the entire feature perception task. The entire feature perception task lasted approximately half an hour.

Throughout both the feature retrieval and perception tasks, key responses were counterbalanced across participants. For instance, one participant might use the left key for the colorful level, while another might use the right key for the same purpose. However, within each participant, the key response assignments remained consistent throughout the experiment. This approach aimed to mitigate the potential switching cost of key responses, which could otherwise prolong reaction times (RT). Our objective was to obtain accurate RT for the feature categorization task and minimize the influence of task‐switching costs. All stimuli were presented using PsychoPy (Peirce et al. 2019).

### 2.4 MEG Data Acquisition

MEG data were acquired using a 306‐sensor (204 planar gradiometers, 102 magnetometers) Elekta Neuromag MEG system (Helsinki, Finland) at Peking University, Beijing, China. Participants completed the MEG experiments inside a sound‐attenuated, dimly lit, and magnetically shielded room. The head position inside the MEG helmet was continuously monitored using four head position indicator (HPI) coils. A 3D digitizer was used to record the location of the HPI coils and the general head shape relative to three anatomical fiducials (nasion, left and right preauricular points). Both horizontal and vertical electrooculograms (EOGs) were recorded. MEG data were recorded at a sampling frequency of 1000 Hz and bandpass filtered between 0.1 and 330 Hz.

### 2.5 The Drift‐Diffusion Model

To explore the evidence accumulation process of three features (color, animacy, and real‐world size) during the perception stage, we used the drift‐diffusion model (DDM) to extract the parameters characterizing the evidence accumulation process of features from the RT. DDMs are widely used for predicting participants' choices and RT during two‐choice decision‐making tasks (Stone 1960; Ratcliff 1978; Ratcliff and McKoon 2008). In the DDM, it is assumed that participants accumulate evidence for one choice over another. The rate of evidence accumulation within a trial is called the drift rate (DR) (δ) (Figure 3A). Evidence is accumulated toward either the upper boundary (e.g., animate) or the lower boundary (e.g., inanimate), corresponding to two respective responses. The boundary separation (α) represents the distance between the two decision boundaries, indicating the amount of evidence needed to make a decision. The starting point (β) reflects the initial position of evidence accumulation and indicates response bias toward one of the two response boundaries. The nondecision time (τ) is the portion of the RT not dedicated to the decision‐making process, typically including preprocessing time and motor response time. The DDM was implemented using the *brms* package in *R* (Bürkner 2017). Only correct trials were used to fit the model. After fitting the model, we extracted the boundary separation, DR, and nondecision time for each task and participant.

### 2.6 MEG Preprocessing

Raw MEG signals were cleaned using the signal space separation (Taulu and Simola 2006) method provided by MaxFilter to suppress magnetic interferences and interpolate bad MEG sensors. MEG data were preprocessed using the MNE toolbox (version 1.1) (Gramfort et al. 2013). To remove line noise, the data were band‐stop filtered at 50 Hz and its harmonics. Muscle artifacts were automatically detected by the MNE function *annotate_muscle_zscore* with default parameters, and the annotations of muscle artifacts were then visually inspected by the experimenters. Signals annotated as muscle artifacts were excluded from further analysis.

Data recorded during the learning stage were epoched between −500 and + 3000 ms relative to the picture onset. During the perception stage, data of feature categorization were epoched between −500 and + 1000 ms relative to the picture onset. The epochs were baseline‐corrected based on the prestimulus signal (−500 ms to onset). During the retrieval stage, data for feature retrieval were epoched between −4000 and + 1000 ms relative to the key response. Since the postresponse signal during retrieval might still contain task‐relevant (i.e., feature‐specific) information, we baseline‐corrected the signal based on the whole trial. The data during the fixation cross before the feature perception (or retrieval) were epoched between −200 and 800 ms relative to the fixation onset. These epochs were baseline‐corrected based on the prefixation signal (−200 ms to onset). Additionally, epochs with excessive noise were discarded using the autoreject toolbox (Jas et al. 2017). Independent component analysis was then used to remove eye‐blink, eye movement, and electrocardiogram (ECG) artifacts. To increase the signal‐to‐noise ratio (SNR) for multivariate decoding, the preprocessed MEG time courses were smoothed using a Gaussian kernel with a full width at a half‐maximum of 10 ms and down‐sampled to 200 Hz.

### 2.7 Time‐Resolved Multivariate Decoding

Before conducting the decoding analysis, we used a smooth window with a 50 ms length and a 10 ms step, averaging signals in each window to further increase the SNR. Our primary focus was on investigating the selective attention modulation effect on feature representations during the perception and retrieval stages. Recognizing that attention may modulate different brain regions in distinct ways, all decoding analyses were carried out separately in four regions of interest (ROIs): the occipital, temporal, parietal, and frontal lobes. The sensors encompassed within each ROI were defined using the MNE toolbox (Gramfort et al. 2013) (Figure S2). While these sensor‐based groupings serve as coarse approximations of the underlying cortical lobes, we acknowledge that MEG sensors are sensitive to signals from both nearby and more distant sources due to volume conduction. Therefore, the anatomical specificity of these ROIs is limited, and signals recorded at a given sensor group may also reflect activity from outside the corresponding lobe.

The learning task functioned as a functional localizer, enabling the training of independent feature classifiers without confounding effects related to motion. For each participant, four distinct classifiers were trained at each time point and ROI using data from the learning stage. These classifiers included one for color category classification (colorful vs. gray), one for animacy category classification (animate vs. inanimate), and two for real‐world size category classification (big vs. small)—one for animate objects and another for inanimate objects (Figure 4A). The reason for classifying the real‐world size separately for animate and inanimate objects was based on research indicating that real‐world size drives differential responses only in the object domain, not the animate domain (Konkle and Caramazza 2013). Therefore, we assumed that the representational patterns of real‐world size for animate and inanimate objects might differ. The feature perception epochs of the same object were averaged. For both the epochs of feature perception and retrieval, the preprocessed amplitudes of channels in each ROI at each time point were whitened and then used as features for classifiers. The whitening process involved using a standard scaler that z‐scored each channel at each time point across trials. Classification using logistic regression models was then performed separately for each time point, ROI, and participant, with a stratified eightfold cross‐validation approach. The logistic regression model parameters were set to their default values as provided by the Scikit‐learn package (King et al. 2016), using L2 regularization, a tolerance for stopping criteria of 1e‐4, an inverse of regularization strength (C) of 1, and the “lbfgs” solver (Limited‐memory Broyden–Fletcher–Goldfarb–Shanno) for optimization. The decoding performance during the learning stage was indicated by the Area Under the Receiver Operating Characteristic Curve (ROC_AUC). We used cluster‐based permutation analyses (Maris and Oostenveld 2007), which intrinsically correct for multiple comparisons, to test the significance of feature representations during the learning stage in each ROI.

After identifying the significant cluster of each feature in each ROI at the group level (averaged across participants) during the learning stage, the time point of peak ROC_AUC within the significant cluster and its surrounding four time points (e.g., if the peak time point was the 78th, the surrounding time points were the 76th, 77th, 79th, and 80th) was considered the most informative time window (i.e., peak bin) carrying the strongest feature information. The averaged and standardized amplitudes in each peak bin were used to train the feature classifiers, which were then tested at each time point in each task during the perception and retrieval stages to detect feature information without any motor confounding effects. For example, the animacy classifier trained during the learning stage in each ROI was tested in the three tasks (the color, animacy, and size tasks) respectively during the perception and retrieval stages in the corresponding ROI. Cross‐validation was not used for across‐stage analyses as no inferences were made based on the decoding performance in the learning stage itself.

To detect the onset and peak times of feature representations during the perception stage, following the approach of the previous study (Linde‐Domingo et al. 2019), we conducted classification analyses at a single trial level. As emphasized by the previous study (Linde‐Domingo et al. 2019), many factors could obscure differences in timing between conditions when averaging latencies across trials, such as variance in processing speed. Therefore, the single‐trial‐based method was considered more sensitive to differences in timing between conditions. We tested the trained classifiers at each time point of each trial, resulting in a total of 16 decoding performance time courses for each participant and each trial: four ROIs (occipital, temporal, parietal, and frontal cortex) by four features (color for all objects, animacy for all objects, and size for inanimate and animate objects, respectively). We used both the peak latency of significant clusters of each trial as the time index to measure the time lag between different features and the onset (i.e., the first time point) of the first significant cluster of each trial. We posited that the onset time signifies the initiation of feature representations, capturing the very inception of when these features begin to manifest in the brain. Detecting this initial time point is crucial for understanding when these features first come into representation. On the other hand, the peak latency signifies the time at which a feature is represented with the highest fidelity. In this context, the onset time allows us to discern whether tasks induce the earlier detection of target features compared to nontarget features in the brain. Meanwhile, the peak time helps us understand whether target features are represented with the highest fidelity before nontarget features. This dual approach provides a comprehensive understanding of the temporal dynamics of feature representations modulated by selective attention.

The decision value (i.e., d‐value) is an appropriate index for characterizing decoding performance at the single‐trial level (Ritchie et al. 2015; Linde‐Domingo et al. 2019). The d‐value has both negative and positive signs, indicating the feature level to which the observation was classified (e.g., − for animate and + for inanimate). The absolute d‐value at each time point represents the distance to the hyper‐plane that divides the two feature levels (e.g., animate and inanimate), with the hyper‐plane being 0 (Figure 4A). This distance indicates the classifier's confidence in assigning a given object to a specific feature level at that time point. For extracting significant clusters carrying feature information for each single trial, the cluster‐based permutation analysis based on multiple trials is not applicable. Therefore, we compared d‐values at each time point in each trial with the threshold of the corresponding time point. A time window of at least T ms, where all the time points surpassed the corresponding thresholds, was considered a significant cluster. Details about null distributions are provided in the next section, “Generating empirical null distributions for classifiers.”

### 2.8 Generating Empirical Null Distributions for Classifiers

To detect the significant clusters of feature representations at the single‐trial level, we followed the method in the previous study (Stelzer et al. 2013; Linde‐Domingo et al. 2019), using permutation and bootstrapping to generate null distributions for each time point of each feature level in each task type. We then chose the 95th or fifth percentile of each null distribution as the threshold for the corresponding time point. In each trial, a time window of at least T ms where all the time points surpassed the corresponding thresholds was considered a significant cluster. The first time point of the first significant cluster was considered the earliest time point when the feature was represented in this trial, that is, the onset time. The time point with the highest d‐value across all significant clusters in each trial was considered the time point when the feature was represented with the highest fidelity, that is, the peak time.

For the permutation analyses, we randomly shuffled the feature level labels (e.g., shuffled the colorful and gray labels) of trials during the learning stage at each permutation and carried out the same training procedure as for the real data. The classifiers were then tested on the data of three tasks during the perception and retrieval stages using the same test procedures as for the real data. This procedure was conducted 150 times independently per participant. For each participant, ROI, task, and feature type, there was a total of 151 classification outputs for each trial of the perception and retrieval stages, respectively: one using the real labels and the remaining using the randomly shuffled labels when training the classifiers. The real data were included to make our subsequent bootstrapping analyses more conservative since, under the null hypothesis, the real classifier output could have been obtained just by chance.

Then the bootstrapping approach was used to estimate the classification chance distribution. For each ROI, task, and feature type, we randomly selected one of the 151 classification outputs per participant in each bootstrapped repetition. Trials in this classification output were divided into two groups based on their feature levels, and the d‐value time courses of each feature level were averaged across trials. As a result, for each ROI, task, and feature type in each bootstrapped repetition, each participant had two d‐value time courses—one feature level each (e.g., one for the animate level and the other for the inanimate level). The d‐value time courses for each feature level were then averaged across participants. This procedure was repeated with replacement 10,000 times. Therefore, for each feature level, each time point had a null distribution consisting of 10,000 values. For levels with positive d‐values (i.e., gray, inanimate, and small), we selected the 95th percentile of each null distribution as the threshold for the corresponding time point. For levels with negative d‐values (i.e., colorful, animate, and big), we selected the fifth percentile of each null distribution as the threshold for the corresponding time point. This decision was made because the absolute value of the fifth percentile would be higher than that of the 95th percentile for negative values, indicating stronger classification confidence. In each trial, a time window of at least T ms where all the time points surpassed the corresponding thresholds was considered a significant cluster. The minimum duration (T) of a significant cluster was tested from 20 to 100 ms with a step size of 10 ms to observe the impact of T on the detected onset times and the relative time lag among different features in each task. Generally, when T was set to 20, 30, and 40 ms, the onset times of all features were within 100 ms, which was consistent with previous research (Cichy et al. 2014; Ritchie et al. 2015; Cichy and Pantazis 2017; Teichmann et al. 2019, 2020, 2021). When T was adjusted to more than 40 ms, the onset times of features exceeded 100 ms. As T increased, the onset times were delayed, and the patterns of the time lags among features, although obscured, remained (examples in Figure S4). Previous empirical research found early onset latencies of decoding accuracy for various categorization levels to be between 48 and 70 ms (Cichy et al. 2014; Ritchie et al. 2015). Among the various values of T we tested, our observed onset times were closest to those reported in previous studies when T was set to 20 ms. Therefore, we determined that a time window of at least 20 ms, where all time points exceeded the corresponding threshold, was considered a significant cluster containing feature information. In each trial, the first time point of the first significant cluster was considered the earliest time point at which the feature was represented. The time point with the highest d‐value across all significant clusters in each trial was considered the peak time point. It should be noted that the peak time within the RT of each trial was considered effective and used for further analysis. Ultimately, for each participant, ROI, and task type, we obtained onset and peak times for color, animacy, and size features for each trial.

### 2.9 Generalized Linear Mixed Model (GLMM) Analyses

Following the previous study (Linde‐Domingo et al. 2019), we employed GLMMs to examine our hypotheses regarding the relative onset or peak time lags of d‐values among different features within the same categorization task during the perception and retrieval stages. We chose GLMMs over the more commonly used GLM‐based models (i.e., ANOVAs or *t* tests) because they make fewer assumptions about the data distribution and are better suited for modeling d‐value onsets. The task type (color, animacy, and size tasks), feature type (color, animacy, and size features), and their interactions were modeled as fixed effects in the GLMM. By examining the interaction between task type and feature type, we assessed whether selective attention modulated the onset or peak times of different features. Participant ID (including the intercept) was modeled as a random factor. All models for analyzing d‐value onset or peak times used a gamma probability distribution and an identity link function, as employed by the previous study (Linde‐Domingo et al. 2019). Unless otherwise specified, *p* values were corrected using Holm–Bonferroni correction for multiple comparisons.

### 2.10 Phase Coupling Estimation

If selective attention can modulate the times of feature representations, how is this process achieved? We hypothesized that during the fixation period (i.e., the task preparation stage) when stimuli (or spatial cues) were not presented, the brain networks were configured to be prepared for the appropriate execution of the instructed task. The stronger the connectivity between brain regions, the more prepared the configured brain networks are, and the faster the target features might be processed. We used the phase slope index (PSI) to characterize the connectivity patterns between the occipital and the other three higher‐order regions (the temporal, parietal, and frontal lobes) and between the temporal and frontal lobes (a control analysis to contrast with the retrieval task) during the preparation stage in the feature perception task. The PSI estimates frequency‐specific phase coupling between brain regions and, crucially, infers the direction of information flow by identifying which of two phase‐coupled areas leads the other (Nolte et al. 2008). We selected PSI for three reasons. First, by focusing on phase‐slope differences rather than absolute phase angles, PSI is robust to volume‐conduction artifacts and yields far fewer false positives than frequency‐domain Granger causality when independent sources are linearly mixed (Nolte et al. 2008). Second, PSI tolerates nonlinear phase spectra, allowing reliable estimation even when the phase–frequency relationship deviates from a strict linear delay. Third, PSI can be applied efficiently across many region pairs and frequency bands in MEG data without the heavy a priori model specification required by Dynamic Causal Modeling (DCM), making it well suited for our whole‐brain, high‐temporal‐resolution analyses.

For the preparation stage in the feature retrieval task, we calculated the PSI between the temporal and frontal lobes. The PSI between two time series for channels *i* and *j* is defined as

$$\tilde{\Psi }\;\mathrm{ij}=I\left(\sum_{f\in F}C_{\mathrm{ij}}^{*}\left(f\right)C_{\mathrm{ij}}\left(f+\mathrm{\delta f}\right)\right)$$

where $C_{\mathrm{ij}}\left(f\right)=S_{\mathrm{ij}}\left(f\right)/\sqrt{S_{\mathrm{ii}}\left(f\right)S_{\mathrm{jj}}\left(f\right)}$ is the complex coherency, *S* is the cross‐spectral matrix, δf is the frequency resolution, and I(·) denotes taking the imaginary part. *F* is the set of frequencies over which the slope is summed. We used the continuous Morlet wavelet transform to estimate the cross‐spectral matrix.

Before calculating PSI, we employed principal component analysis (PCA), also known as spatial filters, to transform the channel data to new sources or virtual channels for each participant and ROI. This step aimed to reduce computation time and increase the SNR. The PCA analysis was conducted separately for the preparation stage in the feature perception and retrieval tasks. The representative time courses in each ROI were defined as the smallest set of signals that explained at least 90% of the raw amplitude of the preprocessed MEG time courses.

PSI values were calculated for all pairs of virtual channels of each pair of ROIs with a frequency resolution of 1 Hz for each frequency band (theta: 4–8 Hz; alpha: 9–13 Hz; beta: 14–30 Hz; gamma: 31–100 Hz). For each pair of ROIs, the higher‐order ROIs were set as seed ROIs, and the occipital lobe was set as the target ROI. For example, participant 1 had *n* virtual channels in the temporal lobe (the seed ROI) and *m* virtual channels in the occipital lobe (the target ROI); the PSI values for the temporal‐occipital pair would form an *n* × *m* matrix for each time point. The sign of PSI values indicates the direction of information transfer. For instance, when the temporal and occipital cortex were set as the seed and target ROIs, respectively, a positive PSI value means the information transfer direction is from the temporal to the occipital lobe, while a negative PSI value indicates the opposite direction. We employed cluster‐based permutation analyses to investigate in which time ranges the PSI values of each task, frequency band, and pair of ROIs differed significantly from zero. This analysis indicated the presence of directional phase coupling between ROIs, helping us understand the neural connectivity patterns that underpin selective attention modulation during the task preparation stages.

After identifying the significant clusters of PSI at the group level for the preparation stage in the feature perception task, we extracted the PSI values from the corresponding time range of significant clusters for each participant and averaged them across the time points in each cluster. When the PSI values of a significant cluster were negative, they were converted to positive values, with larger values indicating stronger phase coupling. If there was more than one significant cluster in a condition, the PSI values were averaged across clusters. We then performed a Pearson's correlation between the averaged PSI values and the onset times of features in the target ROI to investigate whether stronger phase coupling between brain regions during the task preparation stage correlated with earlier onset times of target features instead of nontarget features during the perception stage. For each participant and ROI, the onset time of target features was calculated as the average of the onset times of the color feature in the color task, the animacy feature in the animacy task, and the size feature in the size task. The onset time of nontarget features was calculated as the average of the onset times of the color feature in the animacy and size tasks, the animacy feature in the color and size tasks, and the size feature in the color and animacy tasks.

## 3 Results

### 3.1 Behavioral Performance

**FIGURE 2:**
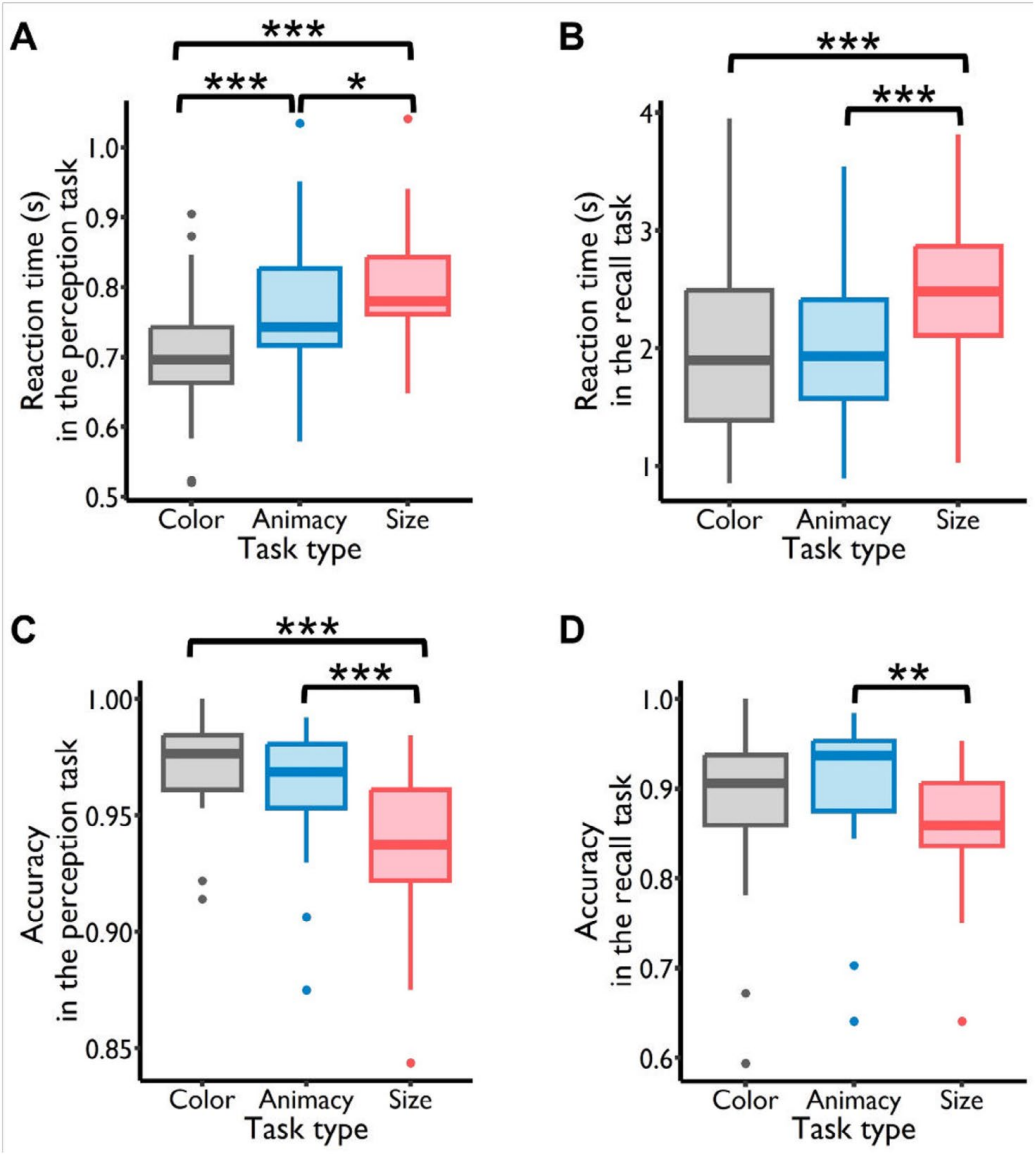
Behavioral results during the perception and retrieval tasks. (A) Analysis of reaction times (RTs) for the color, animacy, and size perception tasks showed a significantly increasing pattern: RT_(color)_ < RT_(animacy)_ < RT_(size)_, with all comparisons being statistically significant. (B) Reaction times in the size retrieval task were significantly longer than in both the color and the animacy retrieval tasks. (C) Accuracy in both color and animacy perception tasks significantly surpassed accuracy in the size task. (D) Accuracy in the animacy retrieval task was significantly higher than in the size retrieval task. *p < 0.05, **p < 0.01, ***p < 0.001.

In the first analysis, we aimed to examine the processing stream of different features through RT. Based on previous findings (Clarke et al. 2013; Linde‐Domingo et al. 2019; Mirjalili et al. 2021), we hypothesized that during the visual perception stage, RT for perceptual features would be faster than those for conceptual features. Only correct trials were used for all RT analyses. Our results aligned with previous research. In the feature perception task, the mean RT were 0.699 s (SD = 0.092 s) for the color task, 0.770 s (SD = 0.104 s) for the animacy task, and 0.798 s (SD = 0.088 s) for the size task. We used a linear mixed‐effects model to directly test the differences in RT across the three tasks. The task type (color, animacy, and size) was used to predict RT, with participant IDs included as a random effect. Task type significantly predicted RT (*F*_2, 52_ = 48.738, *p* < 0.001). Post hoc tests indicated that the RT for the color task was faster compared to both the animacy (*t*_52_ = −6.876, *p*_corrected_ < 0.001) and size tasks (*t*_52_ = −9.574, *p*_corrected_ < 0.001), and RT in the animacy task was faster compared to the size task (*t*_52_ = −2.698, *p*_corrected_ = 0.025) (Figure 2A). In the feature retrieval task, we did not observe reversed patterns for RT compared to the feature perception task. The mean RT were 2.083 s (SD = 0.837 s) for the color task, 2.048 s (SD = 0.682 s) for the animacy task, and 2.508 s (SD = 0.719 s) for the size task. The fixed factor task type significantly predicted RT (*F*_2, 52_ = 16.124, *p* < 0.001). Post hoc testing revealed that the RT for the size task was significantly longer compared to both the color and animacy tasks (size task versus color task: *t*_52_ = 4.713, *p*_corrected_ < 0.001; size task versus animacy task: *t*_52_ = 5.100, *p*_corrected_ < 0.001) (Figure 2B).

Next, we assessed the accuracy in correctly perceiving or retrieving the features in each task type. The accuracy in the feature perception task was close to the ceiling for the color and animacy tasks (color task: Mean = 97.05%, SD = 2.24%; animacy task: Mean = 96.30%, SD = 2.69%), and high for the size task (size task: Mean = 93.58%, SD = 3.15%). The fixed factor task type significantly predicted participants' accuracy (*F*_2, 52_ = 24.435, *p* < 0.001). Post hoc tests indicated that both the color and animacy tasks showed significantly higher accuracy than the size task (color task vs. size task: *t*_52_ = 6.644, *p*_corrected_ < 0.001; animacy task vs. size task: *t*_52_ = 5.205, *p*_corrected_ < 0.001). The accuracy of the color and animacy tasks was comparable (*t*_52_ = 1.440, *p*_corrected_ = 0.328) (Figure 2C). In the feature retrieval task, the mean accuracy was 89.06% (SD = 9.37%) for the color task, 90.39% (SD = 7.99%) for the animacy task, and 86.23% (SD = 7.02%) for the size task. The fixed factor task type significantly predicted participants' accuracy (*F*_2, 52_ = 5.913, *p* < 0.01). Post hoc testing revealed that the accuracy of the animacy task was significantly higher compared to that of the size task (animacy task vs. size task: *t*_52_ = 3.366, *p*_corrected_ < 0.01), while the accuracy of the color task did not exhibit a significant difference compared to the other two tasks (color task vs. animacy task: *t*_52_ = −1.075, *p*_corrected_ = 0.533; color task vs. size task: *t*_52_ = 2.291, *p*_corrected_ = 0.066) (Figure 2D). The behavioral results of the recognition task are presented in Figure S1.

### 3.2 The Evidence Accumulation Process of Three Features

**FIGURE 3:**
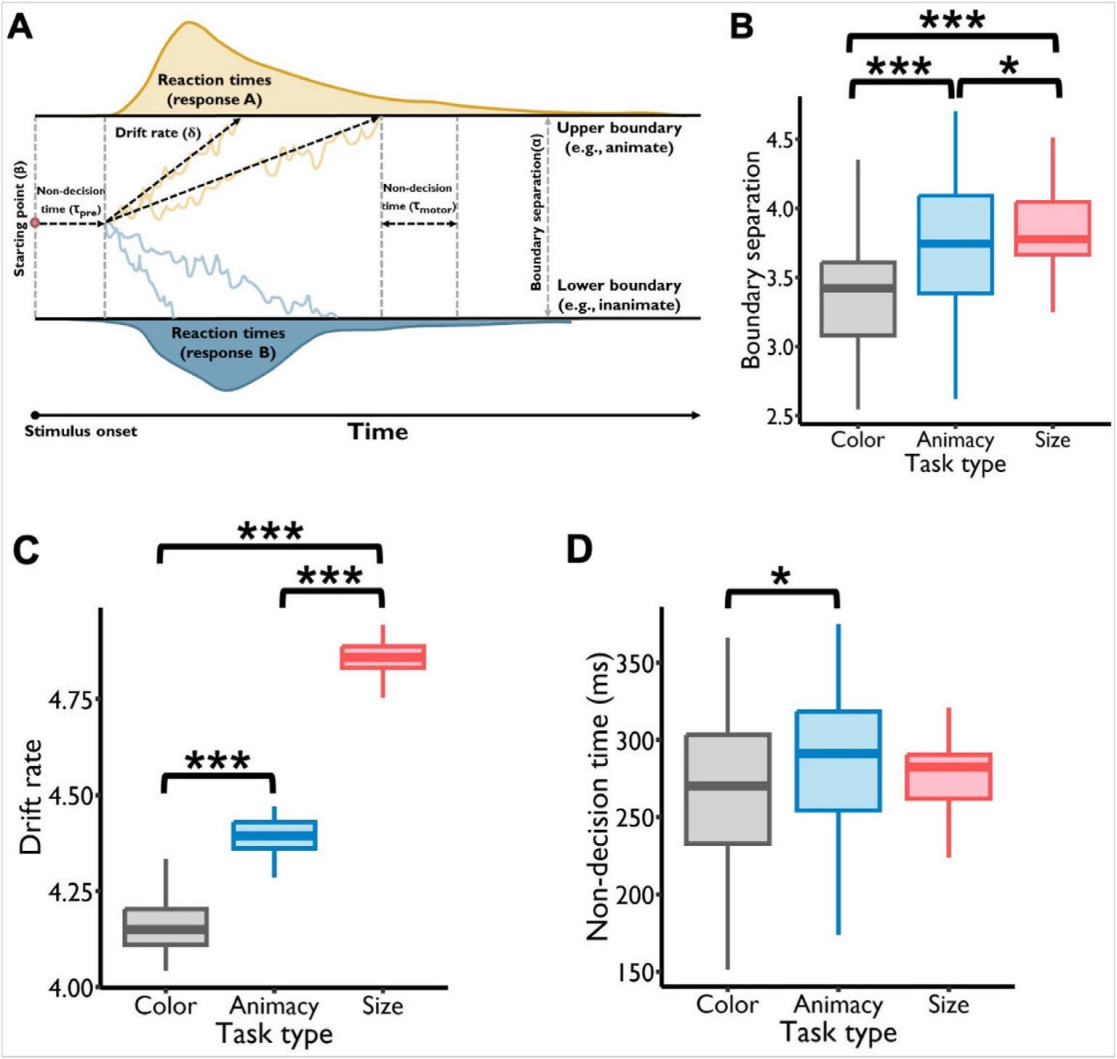
Results of the drift‐diffusion model. (A) Graphical illustration of the drift‐diffusion model. At the onset of a trial, a participant's information level commences at β. Over time, feature information accumulates until it reaches either the upper boundary (e.g., animate) or the lower boundary (e.g., inanimate), resulting in response A or B. The distance between the two decision boundaries is the boundary separation (α). The accumulation rate of feature information is represented by the drift rate δ. The time utilized for preprocessing before evidence accumulation and motor response after decision‐making is the nondecision time τ. (B) Pattern of boundary separation. A discernible pattern showcased increasing boundary separation along the color, animacy, and size tasks, and all of the comparisons were statistically significant. (C) Drift rate. The drift rate (DR) of color, animacy, and size features showed a significantly increasing pattern: DR_(color)_ < DR_(animacy)_ < DR_(size)_, and all of the comparisons were statistically significant. (D) Nondecision time. The nondecision time of the color feature was significantly shorter than that of the animacy feature. *p < 0.05, **p < 0.01, ***p < 0.001.

Behavioral results revealed that RT varied systematically across feature types, with color decisions being the fastest, followed by animacy and size. While these differences in RT suggest a hierarchical order in feature processing, they do not directly inform us about the underlying mechanisms driving these differences. To further investigate whether the observed RT differences stem from variations in evidence accumulation, we applied the DDM (Ratcliff 1978; Ratcliff and McKoon 2008). The DDM allows us to decompose RT into key cognitive components, including DR (δ)—reflecting the speed of evidence accumulation, boundary separation (α)—indicating the amount of evidence required for a decision, and nondecision time (τ)—capturing sensory encoding and motor response delays. Greater boundary separations, longer nondecision time, and slower DRs would lead to slower RT (Figure 3A).

The mean boundary separation for the color, animacy, and size tasks was 3.39 (SD = 0.41), 3.71 (SD = 0.53), and 3.85 (SD = 0.26). A linear mixed‐effects model revealed that task type significantly predicted the boundary separation (*F*_2, 52_ = 46.47, *p* < 0.001). Post hoc testing showed that the boundary separation for the size task was significantly greater than that for the color (*t*_52_ = 9.42, *p*_corrected_ < 0.001) and animacy tasks (*t*_52_ = 2.91, *p*_corrected_ < 0.05), and the boundary separation for the animacy task was significantly greater than that for the color task (*t*_52_ = 6.50, *p*_corrected_ < 0.001) (Figure 3B). This pattern revealed progressively greater decision boundaries for the color, animacy, and size features, indicating that increasingly more evidence needed to be accumulated before a decision was made. This corresponded to the progressively longer RT for these features. In addition, the mean DR for the color, animacy, and size tasks was 4.16 (SD = 0.07), 4.39 (SD = 0.05), and 4.87 (SD = 0.05). A linear mixed‐effects model revealed that task type significantly predicted the DR (*F*_2, 52_ = 3115.56, *p* < 0.001). The DR of color, animacy, and size tasks showed a significantly increasing pattern: DR (color) < DR (animacy) < DR (size), with all comparisons being statistically significant (animacy task vs. color task: *t*_52_ = 25.59, *p*_corrected_ < 0.001; size task vs. color task: *t*_52_ = 77.46, *p*_corrected_ < 0.001; size task vs. animacy task: *t*_52_ = 51.88, *p*_corrected_ < 0.001) (Figure 3C). The mean nondecision time for the color, animacy, and size tasks was 265.47 ms (SD = 48.91 ms), 285.77 ms (SD = 52.06 ms), and 277.72 ms (SD = 23.83 ms). The results of the linear mixed‐effects model showed that task type significantly predicted the nondecision time (*F*_2, 52_ = 3.50, *p* < 0.05). Post hoc testing showed that the nondecision time for the animacy task was significantly longer than that for the color task (*t*_52_ = 2.63, *p*_corrected_ < 0.05). However, there were no significant differences in nondecision time between the color and size tasks (*t*_52_ = −1.59, *p*_corrected_ = 0.26) or between the animacy and size tasks (*t*_52_ = 1.04, *p*_corrected_ = 0.55) (Figure 3D).

These results suggested that the differences in RTs among the three tasks were primarily due to the variations in boundary separation. Additionally, the nondecision time for the animacy feature was longer than that for the color feature, which might also contribute to the longer RTs for the animacy task compared to the color task. This confirmed that greater boundary separations and longer nondecision times contributed to longer behavioral response times. Taken together, the DDM results clarify why conceptual features can yield slower behavioral responses despite a faster accumulation rate—namely, larger decision boundaries (and slightly longer nondecision times) overshadow that advantage. However, these behavioral outcomes alone cannot determine whether conceptual features must invariably be processed after perceptual features in the brain or whether top‐down factors can reorder their onset times. To address this question, we next used MEG decoding to examine how selective attention modulates the timing of perceptual versus conceptual feature representations at the neural level.

### 3.3 Selective Attention Modulated the Onset Times of Feature Perception

We next investigated whether selective attention modulates the timing at which perceptual and conceptual features emerge in the brain. To do so, we combined MEG with multivariate classification analyses, aiming to track the onset and peak times of color, animacy, and size features across different tasks. Based on prior evidence of attentional enhancement, we hypothesized that target (task‐relevant) features would be represented earlier than nontarget features during the perception stage.

**FIGURE 4:**
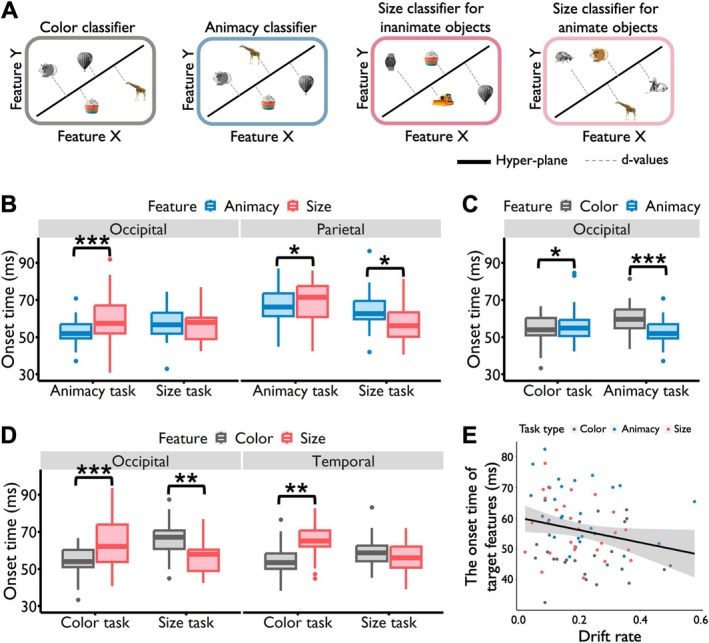
Modulation effects of selective attention during the perception stage. (A) Classifier training. Four classifiers were trained in each ROI using data from the learning stage for each participant. These included one color classifier (colorful vs. gray), one animacy classifier (animate vs. inanimate), and two real‐world size classifiers (small vs. big) for inanimate and animate objects. All classifiers were tested at each time point of each trial in the feature perception and retrieval tasks. (B) Comparing the animacy and size features. In the occipital lobe, the animacy feature had significantly earlier onset times than the size feature during the animacy task, while no significant difference was observed in the size task. Notably, in the parietal lobe, the animacy feature had earlier onset times in the animacy task, whereas the size feature showed earlier onset times in the size task, reflecting a reversed pattern. (C) Comparing the color and animacy features. In the occipital lobe, the color feature exhibited significantly earlier onset times than the animacy feature during the color task. Conversely, in the animacy task, the color feature had significantly later onset times compared to the animacy feature. (D) Comparing the color and size features. Also in the occipital lobe, the color feature had significantly earlier onset times than the size feature in the color task, but significantly later onset times in the size task. Similarly, in the temporal lobe, the color feature showed earlier onset times in the color task, while no significant difference was observed between the two features in the size task. (E) Relationship between behavioral drift rate and neural onset latency for target features. Faster drift rates correlate with earlier parietal onset of target‐feature representations (Spearman *ρ* = −0.25, *p*_corrected_ = 0.027). *p < 0.05, **p < 0.01, ***p < 0.001.

Recognizing that attention may modulate different brain regions in distinct ways, all decoding analyses were carried out separately in four ROIs: the occipital, temporal, parietal, and frontal lobes. The sensors encompassed within each ROI were precisely defined using the MNE toolbox (Gramfort et al. 2013) (Figure S2). We used the learning stage as an independent localizer task to find the most informative time window (i.e., the peak bin) of each feature in each ROI (Liu et al. 2019; Wimmer et al. 2020). For each participant, four distinct classifiers were trained at each time point and ROI using data from the learning stage: one to classify the color category (colorful or gray), one for the animacy category (animate or inanimate), and two for the real‐world size category (big or small)—one for animate objects and another for inanimate objects (Figure 4A). During the learning stage, we found significant clusters of color, animacy, and size features in the occipital, temporal, and parietal lobes (*p*_corrected_ < 0.05). In the frontal lobe, we only found significant clusters of the animacy feature (*p*_corrected_ < 0.05) (Figure S3). It should be noted that significant clusters for the size feature in animate objects were not identified during the learning stage. Consequently, the onset and peak times for the size feature during the perception and recall stages were exclusively calculated for inanimate objects. The peak latencies of significant clusters for the color, animacy, and size features during the learning stage were 305, 515, and 315 ms in the occipital lobe; 365, 485, and 445 ms in the temporal lobe; and 565, 495, and 445 ms in the parietal lobe. The peak latency of the animacy feature in the frontal lobe was 595 ms.

To investigate whether the onset or peak times of target features were earlier than those of nontarget features during the perception and retrieval stages, respectively, we followed a previous study (Linde‐Domingo et al. 2019) in choosing generalized linear mixed‐effect models (GLMMs) over traditional GLM‐based models (e.g., ANOVAs or *t* tests). GLMMs make fewer assumptions about the underlying data distribution and are better suited for modeling single‐trial data, such as RT (Lo and Andrews 2015), including our d‐value onsets and peaks (Linde‐Domingo et al. 2019). They also account for variance explained by both fixed and random variables, including key experimental manipulations. Our conditions of interest were modeled as fixed effects in the GLMM, including the task type (color, animacy, and size tasks), feature type (color, animacy, and size features), and their interactions. Participant ID (including intercept) was modeled as a random factor.

During the perception stage, we found significant interactions between task and feature for onset times in the occipital (*F*_4, 17,104_ = 10.659, *p* < 0.001), temporal (*F*_4, 16,809_ = 6.663, *p* < 0.001), and parietal lobes (*F*_4, 17,066_ = 4.173, *p* = 0.002). Then we examined whether the onset times of (1) conceptual features (i.e., animacy vs. size) or, in a further step, (2) perceptual and conceptual features (i.e., color vs. animacy; color vs. size) were modulated by selective attention. We anticipated that the onset times of target features would precede those of nontarget features. Additionally, if conceptual features were designated as target features, their onset times might precede those of perceptual features when the latter were nontarget features.

For features belonging to the conceptual category, that is, the animacy and size features, post hoc analyses showed that in the occipital lobe, the onset times of the animacy feature (target feature) were significantly earlier than those of the size feature (nontarget feature) in the animacy task (animacy feature vs. size feature: *t*_17,104_ = −4.019, *p*_corrected_ < 0.001). However, in the size task, the onset times of these two features did not show a significant difference (animacy feature vs. size feature: *t*_17,104_ = −0.013, *p*_corrected_ = 0.989) (Figure 4B). More importantly, in the parietal lobe, the onset times of the animacy feature were significantly earlier than those of the size feature in the animacy task (animacy feature vs. size feature: *t*_17,066_ = −1.969, *p*_corrected_ = 0.049). Conversely, a reversed pattern was found in the size task (animacy feature vs. size feature: *t*_17,066_ = 2.352, *p*_corrected_ = 0.019) (Figure 4B). These results showed that selective attention made the onset times of the target feature occur earlier than those of the nontarget feature.

For features belonging to different categories, that is, the perceptual feature color and the conceptual feature animacy, post hoc comparisons indicated that in the occipital lobe, the onset times of the color feature were significantly earlier than those of the animacy feature in the color task (color feature vs. animacy feature: *t*_17,104_ = −2.114, *p*_corrected_ = 0.035). Conversely, in the animacy task, the onset times of the color feature were significantly later than those of the animacy feature (color feature vs. animacy feature: *t*_17,104_ = 4.901, *p*_corrected_ < 0.001) (Figure 4C). These findings indicate that selective attention indeed influenced the onset times of both perceptual and conceptual features.

A similar pattern was observed for the color and size features. Also, in the occipital lobe, the onset times of the color feature occurred significantly earlier than those of the size feature in the color task (color feature vs. size feature: *t*_17,104_ = −3.930, *p*_corrected_ < 0.001), while in the size task, the onset times of the color feature occurred significantly later than those of the size features (color feature vs. size feature: *t*_17,104_ = 2.916, *p*_corrected_ = 0.004) (Figure 4D). We also found that in the temporal lobe, the onset times of the color feature were significantly earlier than those of the size feature in the color task (color feature vs. size feature: *t*_16,809_ = −2.956, *p*_corrected_ = 0.003), while in the size task, the onset times of the color and size features did not differ (color feature vs. size feature: *t*_16,809_ = 1.138, *p*_corrected_ = 0.255) (Figure 4D). These results highlight the important role of selective attention in modulating the onset times of features at different levels. Intriguingly, selective attention even led to earlier onset times for conceptual features compared to perceptual features during the perception stage.

To relate behavioral evidence accumulation to neural timing, we correlated each participant's DR with onset latencies. Target features—that is, the feature currently being judged (color in the color task, animacy in the animacy task, size in the size task)—showed a significant negative relationship in parietal cortex (Spearman *ρ* = −0.25, *p*_corrected_ = 0.027), indicating that faster evidence accumulation predicted earlier neural onset (Figure 4E). No such relationship was observed for the same features when they were nontargets in other tasks (*ρ* = −0.17, *p*_corrected_ = 0.124). This brain–behavior correlation serves as a validity check, confirming that both our DDM and our neural index capture the underlying evidence‐accumulation process.

### 3.4 Phase Coupling Strength During Task Preparation Correlated With Earlier Onset of Feature Perception

**FIGURE 5:**
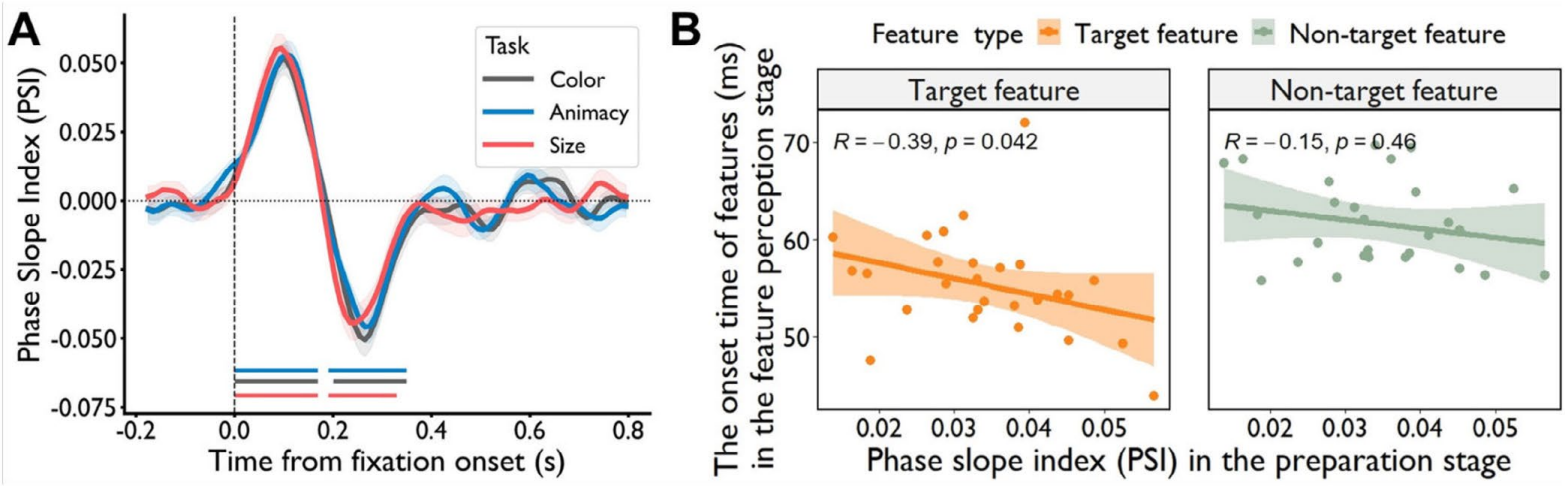
Phase coupling results in the feature perception task. (A) Phase coupling patterns during the task preparation stage. During the task preparation stage, defined as the fixation period before image presentation, significant phase coupling in the theta band was observed between the temporal and occipital lobes across all task types (*p*_corrected_ < 0.001). Two distinct directions of coupling were identified: one from the temporal to the occipital lobe, and the other in the opposite direction. (B) Correlation with onset times. A significant negative correlation was observed across subjects between the strength of temporal‐occipital phase coupling in the theta band during the task preparation stage and the onset times of target features (*R* = −0.39, *p*_corrected_ = 0.042), but not of nontarget features (*R* = −0.15, *p*_corrected_ = 0.46), in the occipital lobe during perception.

Selective attention accelerated target‐feature onsets most strongly in the occipital lobe. To test whether this effect was driven by preparatory top‐down signals, we analyzed phase coupling between the occipital lobe and higher‐order regions during the fixation period preceding stimulus onset, using the PSI across multiple frequency bands. In this analysis, the occipital lobe served as the target ROI, with the higher‐order regions acting as seed ROIs. We then examined the relationship between the strength of this phase coupling during the task preparation stage and the onset times of feature representations in the occipital lobe during the perception stage. Our results revealed that the strength of phase coupling between the temporal and occipital lobes in the theta band during the task preparation significantly correlated with the onset times of target features, but not nontarget features during the perception stage. Specifically, two significant clusters were identified in the theta band for each task type (*p*_corrected_ < 0.001) (Figure 5A). In each task type, PSI values in the first significant cluster were positive, indicating a top‐down flow of information from the temporal to the occipital lobe. Conversely, PSI values in the second significant cluster were negative, signifying a reversed information flow compared to the first cluster. This suggested that, in the theta band, the information flow between the temporal and occipital cortex followed a sequential top‐down and bottom‐up process during the task preparation stage. The phase coupling patterns between the temporal and occipital lobes across other frequency bands are illustrated in Figure S5.

Having obtained the phase coupling between the seed and target ROIs, we aimed to explore whether phase coupling strength during the task preparation stage facilitated faster recognition of target features compared to nontarget features. To investigate this, we computed Pearson's correlation between phase coupling strength during task preparation and the onset times of both target and nontarget features in the occipital lobe during the perception stage. Our findings revealed a significant negative correlation between theta band temporal‐occipital phase coupling strength and the onset times of target features (*R* = −0.39, *p*_corrected_ = 0.042; Figure 5B, left panel), whereas no such significant correlation was observed for nontarget features (*R* = −0.15, *p*_corrected_ = 0.46; Figure 5B, right panel) in the occipital lobe. This suggests that a stronger phase coupling between the temporal and occipital lobes in the theta band during the task preparation stage may facilitate faster recognition of target features in the occipital lobe during the subsequent perception stage.

### 3.5 Selective Attention Modulated the Peak Times of Feature Retrieval

The previous analysis unveiled that selective attention modulated the onset times of feature perception, even leading to earlier onset times for conceptual features compared to perceptual features. In a subsequent analysis, we aimed to investigate whether selective attention could also influence the onset or peak times of feature representations during the memory retrieval stage. This inquiry is intriguing because visual perception and memory retrieval are distinct mental processes. Visual perception involves both outside‐in and top‐down processes, where individuals process visual inputs from the external world while also integrating expectations and predictions. In contrast, memory retrieval is primarily an inside‐out process, involving the recall of information stored in the mind. Additionally, past research has indicated that conceptual information is reconstructed faster than perceptual details during the memory retrieval stage, showcasing an inverted pattern compared to the object recognition process (Linde‐Domingo et al. 2019; Mirjalili et al. 2021). The question of how selective attention's modulation effects on feature representational timing vary across these two different mental processes remains unclear. Using the same decoding and statistical analysis methods as in the feature perception task, our findings revealed that selective attention modulated the peak times of feature retrieval rather than the onset times. Specifically, results from the GLMM indicated that the interaction between task type (color, animacy, and size tasks) and feature type (color, animacy, and size features) was significant in the frontal lobe (*F*_4, 8674_ = 5.697, *p* < 0.001), with a trend‐level effect observed in the temporal lobe (*F*_4, 8611_ = 2.189, *p* = 0.068).

**FIGURE 6:**
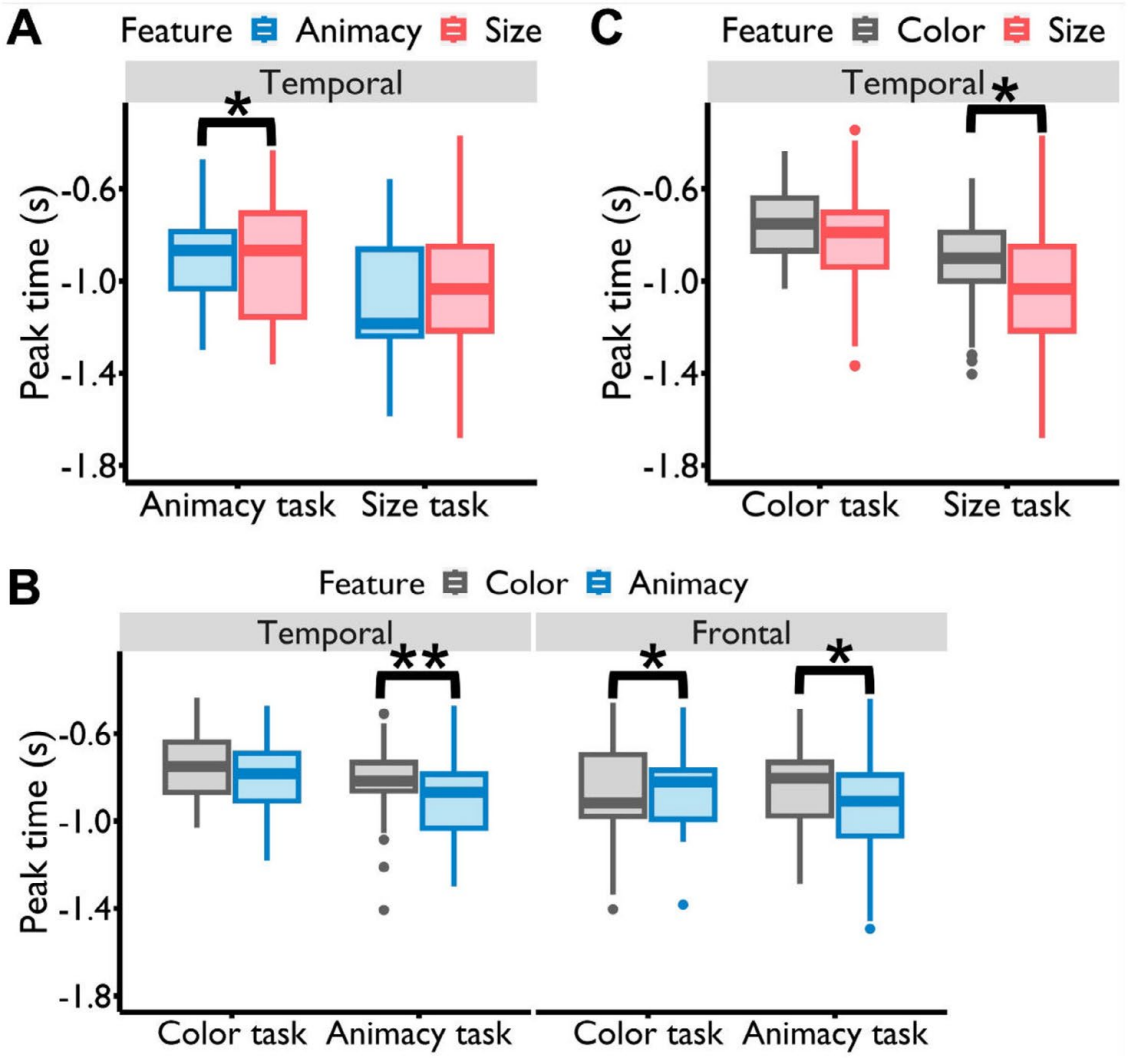
Modulation effects of selective attention on the peak times of feature retrieval. (A) Comparing the animacy and size features. In the animacy task, the peak time of the animacy feature in the temporal lobe occurred significantly earlier than that of the size feature. However, in the size task, the peak times of these two features showed no significant difference. (B) Comparing the color and animacy features. In the frontal lobe, a reverse effect was observed: In the color task, the peak time of the color feature was significantly earlier than that of the animacy feature, while in the animacy task, this pattern was reversed. In the temporal lobe, during the animacy task, the peak time of the animacy feature was significantly earlier than that of the color feature. However, in the color task, no significant difference was observed in the peak times of these two features. (C) Comparing the color and size features. In the size task, the peak time for the size feature in the temporal lobe was significantly earlier than for the color feature, while in the color task, the peak times for these two features exhibited no significant differences. *p < 0.05, **p < 0.01, ***p < 0.001.

For the conceptual features (animacy and size), post hoc comparisons indicated that in the temporal lobe, the peak times of the animacy feature were significantly earlier than those of the size feature in the animacy task (animacy feature vs. size feature: *t*_8611_ = −3.128, *p*_corrected_ = 0.046), while the peak times of these two features showed no significant difference in the size task (animacy feature vs. size feature: *t*_8611_ = −0.996, *p*_corrected_ = 0.986) (Figure 6A). For the perceptual feature color and conceptual feature animacy, post hoc comparisons indicated that in the frontal lobe, the peak time of the color feature was significantly earlier than that of the animacy feature in the color task (color feature vs. animacy feature: *t*_8674_ = −3.414, *p*_corrected_ = 0.019), while this pattern was reversed in the animacy task (animacy feature vs. color feature: *t*_8674_ = −3.121, *p*_corrected_ = 0.047) (Figure 6B). These results indicated that the perceptual feature (color) can be reconstructed with the highest fidelity earlier than the conceptual feature (animacy) in the frontal lobe. In the temporal lobe, the peak time of the animacy feature was significantly earlier than that of the color feature in the animacy task (animacy feature vs. color feature: *t*_8611_ = −3.708, *p*_corrected_ = 0.007), while in the color task, the peak times of these two features did not show a significant difference (animacy feature vs. color feature: *t*_8611_ = −1.430, *p*_corrected_ = 0.886) (Figure 6B). For the color and size feature in the temporal cortex, the peak time of the size feature was significantly earlier than that of the color feature (size feature vs. color feature: *t*_8611_ = −3.455, *p*_corrected_ = 0.016), while there was no significant difference in the peak times between these two features in the color task (size feature vs. color feature: t_8611_ = −1.885, *p*_corrected_ = 0.624) (Figure 6C).

In contrast to the perception stage, our observations during the retrieval stage indicated that attention modulated the peak times rather than the onset times of feature representations. Notably, this modulation resulted in the target features being reconstructed with the highest fidelity earlier than the nontarget features. The comparison between color and animacy features further suggested that attention modulations facilitated a faster reconstruction of perceptual features with the highest fidelity compared to conceptual features.

Intriguingly, in contrast to the feature perception task where modulation effects primarily manifested in the occipital lobe, the feature retrieval task revealed modulation effects predominantly in the temporal and frontal lobes. We posited that during the task preparation stage, participants likely engaged in a preemptive objects or features retrieval, so as to facilitate rapid response upon the spatial cue's appearance. Since modulation effects were observed in the temporal and frontal lobes, we questioned whether synchronized activity between these regions might begin as early as the task preparation stage, helping the brain enter a preparatory state for the upcoming task.

**FIGURE 7:**
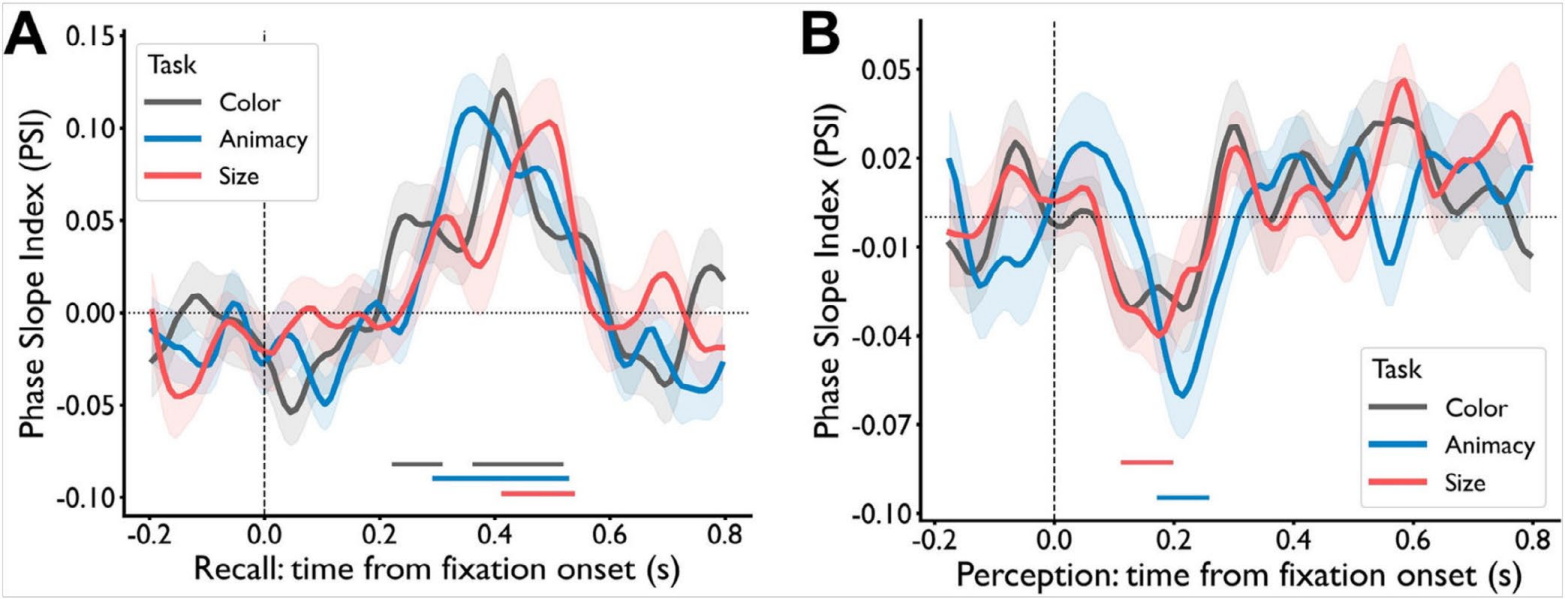
Phase coupling between temporal and frontal lobes in the beta band during the preparation stage. (A) In the feature retrieval task, a significant cluster of phase coupling from the frontal to the temporal lobe was observed in each task type (color, animacy, and size task) (*p*_corrected_ < 0.05). (B) In the feature perception task, a significant cluster of phase coupling from the temporal to the frontal lobe was found in the animacy and size tasks (*p*_corrected_ < 0.05).

To scrutinize interactions between these lobes, we computed the PSI across four frequency bands between the temporal (target ROI) and frontal (seed ROI) lobes, utilizing fixation period data before the spatial cue stage. Remarkably, significant clustering between these regions emerged in the beta band for each task type (color task: 225–305 ms, 365–515 ms; animacy task: 295–525 ms; size task: 415–535 ms) (*p*_corrected_ < 0.05) (Figure 7A). The values in each significant cluster were positive, signifying a prevailing direction of information flow from the frontal to the temporal lobe. A striking departure from this pattern became evident when scrutinizing the results of the perception preparation stage (Figure 7B). Notably, during this stage, no significant cluster featuring the same direction of information flow emerged as was seen in the retrieval preparation stage. Instead, we uncovered a significant cluster with information flow directed from the temporal to the frontal lobe, observed in the animacy (175–255 ms) and size task (115–195 ms) (*p*_corrected_ < 0.05). These nuanced results underscore the specificity of the frontal‐to‐temporal information flow during the task preparation stage of the retrieval task rather than the perception task.

## 4 Discussion

The question of whether information flows through a fixed hierarchical order across different feature levels during visual perception and retrieval stages remains an intriguing topic. Our findings indicate that the timing of perceptual and conceptual features is not rigid but can be flexibly shaped by selective attention. Specifically, during the visual perception, conceptual features can emerge earlier than perceptual ones, whereas during memory retrieval, perceptual features can be reconstructed with the highest fidelity before conceptual features. These results highlight how attentional modulation can override or reverse presumed processing hierarchies and offer new insights into how feature detection and reconstruction are differentially orchestrated in perception and memory.

Using the DDM (Ratcliff 1978; Ratcliff and McKoon 2008), we observed a progressive increase in boundary separation for color, animacy, and size features, mirroring their RT. Although animacy and real‐world size are both classified as conceptual, it is important to recognize that animacy often relies on rapidly processed perceptual cues such as shape or biological motion (Thorat et al. 2019). In contrast, real‐world size typically requires higher‐level inferential reasoning, drawing heavily on semantic knowledge and contextual associations (Konkle and Caramazza 2013). We acknowledge that perceptual cues can, in principle, contribute to size judgments (e.g., canonical part structure or depiction conventions), but in our paradigm such cues were minimized via stimulus counterbalancing, and the longer RTs together with larger boundary separations for size suggest that any purely perceptual contribution was modest. This pattern reinforces the view of a representational continuum—from low‐level perceptual (color), through mid‐level categorical (animacy), to highly conceptual (size).

More importantly, the model result is consistent with theoretical models positing that object information at multiple categorization levels is processed simultaneously but follows distinct evidence accumulation trajectories (Mack and Palmeri 2011; Kravitz et al. 2013). Empirical studies likewise demonstrate early onset latencies for decoding across various categorization levels (48–70 ms), with higher‐level features peaking later than lower‐level features (Carlson et al. 2013; Cichy et al. 2014).

To further explore whether the temporal dynamics of perceptual and conceptual feature perception can be modulated by selective attention, we employed single‐trial‐based decoding analysis, a highly sensitive method for detecting representational times (Linde‐Domingo et al. 2019). As emphasized by the previous research (Linde‐Domingo et al. 2019), many factors can obscure differences in timing between conditions when averaging decoding traces across trials and subjects. For instance, there was substantial variance in RT among participants. Similarly, there were significant variances across trials within the same subject, as factors such as retrieval speed, mental state, and other conditions fluctuate across trials. Averaging decoding traces across trials and subjects would reduce the effective feature information, making it difficult to detect timing differences between conditions. Therefore, a single‐trial‐based method was considered to be more sensitive in capturing such differences. Based on this method, our results revealed that in the visual perceptual task, directing attention to perceptual features (e.g., color) resulted in their onset times preceding those of conceptual features. Interestingly, when attention was shifted to conceptual features (e.g., animacy and real‐world size), the onset times of these conceptual features significantly preceded those of perceptual features (see Figure 4). These findings suggest that selective attention enables the earlier detection of target features over nontarget features. Furthermore, in conceptual tasks, attention can orchestrate a scenario where conceptual features took precedence over perceptual features.

Numerous studies have demonstrated the modulation effects of selective attention on various aspects of information processing and brain responses, including activation strength (Maunsell and Treue 2006; Baldauf and Desimone 2014), representational fidelity (Xue et al. 2013; Lu et al. 2015; Long and Kuhl 2018; Zheng et al. 2018; Grootswagers et al. 2021), dimensions (Sheng et al. 2022), and distance (Nastase et al. 2017). Our findings on the adjustable onset times of perceptual and conceptual feature perception underscore the dynamic and flexible nature of visual perception from a new perspective. This challenges the notion of a fixed sequence where perceptual features invariably precede conceptual features (Clarke et al. 2013; Linde‐Domingo et al. 2019; Mirjalili et al. 2021). These results also contribute to the intense debates on the nature of perception, which center on passive (purely bottom‐up) versus active accounts (Treisman 1980; Firestone and Scholl 2016). Notably, theories of active sensing, dating back to Gibson's seminal work in the 1950s, propose that perception is not merely a passive reception of sensory inputs but is fundamentally driven by internal goals and motivations (Gibson 1950; O'Regan and Noë 2001; Summerfield and De Lange 2014). According to these active accounts, task demands and attention play crucial roles in shaping how information is processed and represented in the brain (Desimone and Duncan 1995; Engel et al. 2001; Nobre and Kastner 2014). This perspective suggests that our perceptual experiences are not solely dictated by external stimuli but are also significantly influenced by our cognitive states and objectives.

It is noteworthy that the modulation effect of selective attention was observed in the occipital lobe for all three feature pairs during the visual perception stage. Previous research has shown that top‐down factors can influence stimulus representations in early visual areas (Jehee et al. 2011; Sprague and Serences 2013; Xue et al. 2013; Baldauf and Desimone 2014; Lu et al. 2015; Ester et al. 2016; Zheng et al. 2018; Sheng et al. 2022). Moreover, cognitive intentions, such as expectations and mental readiness embedded in top‐down processes, may precede the presentation of a stimulus. This intentional processing guides bottom‐up sensory processing by reallocating attention based on subjective expectations, thereby enhancing the efficiency of perceptual identification (Sakai 2008; Siegel et al. 2008; Baldauf and Desimone 2014; Córdova et al. 2016). Our findings replicate and extend these studies by demonstrating that selective attention can modulate the speed of feature accumulation, thereby influencing the temporal dynamics of visual perception.

To understand how this modulation was achieved, we explored the phase coupling between the occipital lobe and higher‐order brain regions during the task preparation stage, when stimuli were not presented. In the theta band, we observed an initial information flow from the temporal to occipital lobe during the first 200 ms, followed by a backward information flow in the subsequent 200 ms. Further analysis revealed that the strength of phase coupling in this initial top‐down and subsequent bottom‐up cluster during the task preparation stage correlated with the onset times of target features, rather than nontarget features, in the occipital lobe during the perception stage. Specifically, stronger phase coupling between the occipital and temporal lobes in the theta band was associated with earlier onset times of target features but not nontarget features. This finding emphasizes the role of interregional interactions during the preparation stage in promoting the speed of evidence accumulation for target features in visual perception tasks.

In addition to perception, our results demonstrated that selective attention could modulate evidence accumulation during retrieval so that conceptual features do not always peak earlier than perceptual features (Linde‐Domingo et al. 2019; Mirjalili et al. 2021). We observed a reversal of the peak times for perceptual and conceptual feature retrieval in the frontal lobe, highlighting the significant role of frontal regions in selective attention. Consistent with our findings, previous research has emphasized the crucial role of frontal regions in prioritizing items in visual working memory, selecting and integrating task‐relevant features, and maintaining working memory flexibility while avoiding interference (Nobre et al. 2004; Nee and Jonides 2008; Chatham et al. 2014; Buschman and Kastner 2015).

In addition to the frontal lobe, we found that the temporal lobe also exhibits a selective attention modulation effect, suggesting that this effect extends to lower‐level regions in the processing hierarchy. Previous research has shown that the frontal cortex, especially the prefrontal cortex, has reciprocal anatomical connections with the inferior temporal cortex (ITC) (Gerbella et al. 2010; Saleem et al. 2014). These connections exert top‐down signals that modulate the visual neural responses of the ITC (Fuster et al. 1985) and induce activity related to memory retrieval (Tomita et al. 1999; Zhou et al. 2023). Our phase coupling analysis revealed a significant positive cluster in the beta band between the frontal (seed ROI) and temporal (target ROI) lobes, indicating information flow from the frontal to the temporal lobe. Notably, this directional information flow was absent during the preparation stage in the feature perception task, indicating task‐specific information flow. These findings illustrate that the brain engages in distinct preparatory processes depending on the task type, whether it is a feature perception or retrieval task. These differences manifest in various ways, including the regions involved in phase coupling and the frequency bands through which information is communicated.

Our study also found that selective attention modulates the onset time of feature representations during the perception stage, whereas it modulates the peak time of feature representations during memory retrieval. Previous research has shown that when an object is recalled, its binding features are often retrieved spontaneously, even when participants are not explicitly instructed to recall those features (Linde‐Domingo et al. 2019; Bone et al. 2020). Because these features are retrieved automatically during recall, this may make selective attention less capable of modulating the onset time than the peak time of their reactivation. In contrast, during feature perception, selective attention can modulate the onset times because individual features can be represented before the object is recognized. Future research is required to better understand the role of selective attention in modulating the cognitive and neural processes across different stages.

There are several points that warrant further exploration. First, although MEG provides excellent temporal resolution, its spatial resolution is limited compared to other neuroimaging techniques like fMRI. Future research could conduct parallel MEG and fMRI studies to gain more detailed insights into both the temporal and spatial dynamics of the modulation effect of selective attention on feature processing. Second, while our PSI analysis revealed directional information flow between brain regions during task preparation, PSI cannot fully disambiguate indirect pathways or model the synaptic mechanisms that DCM can. Complementary approaches—including model‐based analyses (e.g., DCM) and causal perturbation techniques such as transcranial magnetic stimulation or transcranial alternating‐current stimulation—will be important to verify whether the preparatory top‐down influence we observe is necessary for accelerating evidence accumulation. Finally, future studies could utilize computational models to further understand the role of selective attention in modulating the processing hierarchy.

To conclude, this study suggests that perceptual and conceptual features are processed in parallel, and selective attention modulates the temporal dynamics of their processing during both perception and memory retrieval tasks. This modulatory effect is correlated with the phase coupling between the temporal and occipital lobes in the theta band during perception and between the frontal and temporal lobes in the beta band during retrieval. These results underscore the dynamic and flexible nature of attentional modulation in cognitive processing.

## Author Contributions

**Yu Zhou:** conceptualization, formal analysis, investigation, methodology, visualization, writing – original draft, writing – review and editing. **Liang Zhang:** methodology. **Nikolai Axmacher:** writing – review and editing. **Daniel Pacheco Estefan:** writing – review and editing. **Dahui Wang:** investigation. **Yujian Dai:** methodology. **Xiaojing Peng:** investigation. **Shixiang Liu:** conceptualization, writing – review and editing. **Gui Xue:** conceptualization, funding acquisition, investigation, methodology, supervision, writing – review and editing.

## Ethics Statement

The study was approved by the Research Ethics Committee of Peking University and the State Key Laboratory of Cognitive Neuroscience and Learning at Beijing Normal University, China (Approval No. 202009‐29).

## Consent

This study involved healthy adult volunteers (no patients); written informed consent was obtained from all participants.

## Conflicts of Interest

The authors declare no conflicts of interest.

## Supporting information


**Figure S1:** The behavioral results in the recognition task. (A) Reaction time. The mean reaction times of the object recognition under the color, animacy, and size task conditions were 1.732 s (SD = 0.403 s), 1.766 s (SD = 0.402 s), and 1.822 s (SD = 0.412 s). The task type predicted the reaction times (*F*_2, 52_ = 4.255, *p* = 0.019). Post hoc testing showed that the reaction time of the object recognition under the color task condition was significantly faster than that under the size task condition (*t*_52_ = −2.889, *p*_corrected_ = 0.015), while the difference between the animacy task condition and the other two task conditions was not significant (animacy condition vs. color condition: *t*_52_ = 1.097, *p*_corrected_ = 0.520; animacy condition vs. size condition: *t*_52_ = −1.793, *p*_corrected_ = 0.182). (B) Accuracy. The mean accuracy of the object recognition under the color, animacy and size task conditions was 92.59% (SD = 5.44%), 91.38% (SD = 5.12%), and 91.55% (SD = 6.21%). The task type did not predict the accuracy of object recognition under three task conditions (*F*_2, 52_ = 1.181, *p* = 0.315).
**Figure S2:** Elekta Neuromag MEG channel positions. Channels corresponding to different lobes are color‐coded (figure adapted from www.megwiki.org).
**Figure S3:** The decoding performance of three features during the learning stage. Significant clusters of color, animacy and size features in the occipital, temporal and parietal cortex were found (*p*_corrected_ < 0.05). In the frontal cortex, only significant clusters of the animacy feature were found (*p*_corrected_ < 0.05). There were no significant clusters of size feature for the animate objects. The shaded areas surrounding the classification performance time courses indicated standard error across participants. The vertical shaded areas marked the significant clusters carrying the feature information.
**Figure S4:** The modulation effects of selective attention when setting T = 30 ms (A) or 40 ms (B), that is, the time window of at least 30 or 40 ms in which the d‐values of all time points exceeding the corresponding threshold was considered as a significant cluster containing feature information. As shown in the figures, when the minimum duration (T) of a significant cluster increased, the overall onset times of features were delayed accordingly, and the patterns of time lags between different features remained discernible, although some of them became obscured.
**Figure S5:** Phase coupling patterns between the temporal and occipital lobes across different frequency bands. Only phase coupling in the theta band demonstrated a pattern of initially top‐down followed by bottom‐up interactions. In contrast, the phase couplings in the alpha, beta, and low gamma bands were predominantly bottom‐up, indicating an information flow from the occipital to temporal lobes. No significant phase coupling was observed in the high gamma band.
**Figure S6:** Selective‐attention modulation of feature latencies during the perception task. (A) Onset latencies. Animacy versus size. Occipital ROI: animacy onsets were earlier than size onsets in the Animacy task, whereas no reliable difference appeared in the Size task. Parietal ROI: the pattern reversed—animacy led in the Animacy task, but size led in the Size task. Color versus animacy. Occipital ROI: color onsets preceded animacy in the Color task but were delayed relative to animacy in the Animacy task. Color versus size. Occipital ROI: color onsets preceded size in the Color task yet lagged behind size in the Size task. Temporal ROI: color led size in the Color task, with no significant difference in the Size task. (B) Peak latencies. No significant attentional modulation was observed for peak latencies with any feature pair during visual perception. Significance: *p < 0.05, **p < 0.01, ***p < 0.00 (FDR‐corrected). ROI = region of interest.
**Figure S7:** Selective‐attention modulation of feature latencies during memory retrieval. (A) Peak latencies. Animacy versus size. Temporal ROI: animacy peaks occurred earlier than size peaks in the Animacy task; no difference in the Size task. Color versus animacy. Frontal ROI: a bidirectional effect—color peaked earlier than animacy in the Color task, whereas animacy peaked earlier than color in the Animacy task. Temporal ROI: animacy peaked earlier than color in the Animacy task; no difference in the Color task. Color versus size. Temporal ROI: size peaked earlier than color in the Size task; no difference in the Color task. (B) Onset latencies. No significant attentional modulation was detected for onset latencies with any feature pair during memory retrieval. Significance: *p < 0.05, **p < 0.01, ***p < 0.00 (FDR‐corrected). ROI = region of interest.

## Data Availability

The data that support the findings of this study are available from the corresponding author upon reasonable request.
