# Supplementary material for: Selective Attention Dynamically Modulates the Hierarchical Order of Perceptual and Conceptual Representations
Source: Hum Brain Mapp. 2025 Sep 20;46(14):e70359. doi: 10.1002/hbm.70359 (PMC12449827; doi:10.1002/hbm.70359)
Supplement: Supplementary file 1 — Figure S1: The behavioral results in the recognition task. (A) Reaction time. The mean reaction times of the object recognition under the color, animacy, and size task conditions were 1.732 s (SD = 0.403 s), 1.766 s (SD = 0.402 s), and 1.822 s (SD = 0.412 s). The task type predicted the reaction times (F 2, 52 = 4.255, p = 0.019). Post hoc testing showed that the reaction time of the object recognition under the color task condition was significantly faster than that under the size task condition (t 52 = −2.889, p corrected = 0.015), while the difference between the animacy task condition and the other two task conditions was not significant (animacy condition vs. color condition: t 52 = 1.097, p corrected = 0.520; animacy condition vs. size condition: t 52 = −1.793, p corrected = 0.182). (B) Accuracy. The mean accuracy of the object recognition under the color, animacy and size task conditions was 92.59% (SD = 5.44%), 91.38% (SD = 5.12%), and 91.55% (SD = 6.21%). The task type did not predict the accuracy of object recognition under three task conditions (F 2, 52 = 1.181, p = 0.315). Figure S2: Elekta Neuromag MEG channel positions. Channels corresponding to different lobes are color‐coded (figure adapted from www.megwiki.org). Figure S3: The decoding performance of three features during the learning stage. Significant clusters of color, animacy and size features in the occipital, temporal and parietal cortex were found (p corrected < 0.05). In the frontal cortex, only significant clusters of the animacy feature were found (p corrected < 0.05). There were no significant clusters of size feature for the animate objects. The shaded areas surrounding the classification performance time courses indicated standard error across participants. The vertical shaded areas marked the significant clusters carrying the feature information. Figure S4: The modulation effects of selective attention when setting T = 30 ms (A) or 40 ms (B), that is, the time window of at least 30 or [file HBM-46-e70359-s001.docx]

Supplementary Materials for

**Selective Attention Dynamically Modulates the Hierarchical Order of Perceptual and Conceptual Representations**

# Yu Zhou *et al.*

*Corresponding author. Email: [gxue@bnu.edu.cn](mailto:gxue@bnu.edu.cn), [liusx@buu.edu.cn](mailto:liusx@buu.edu.cn)


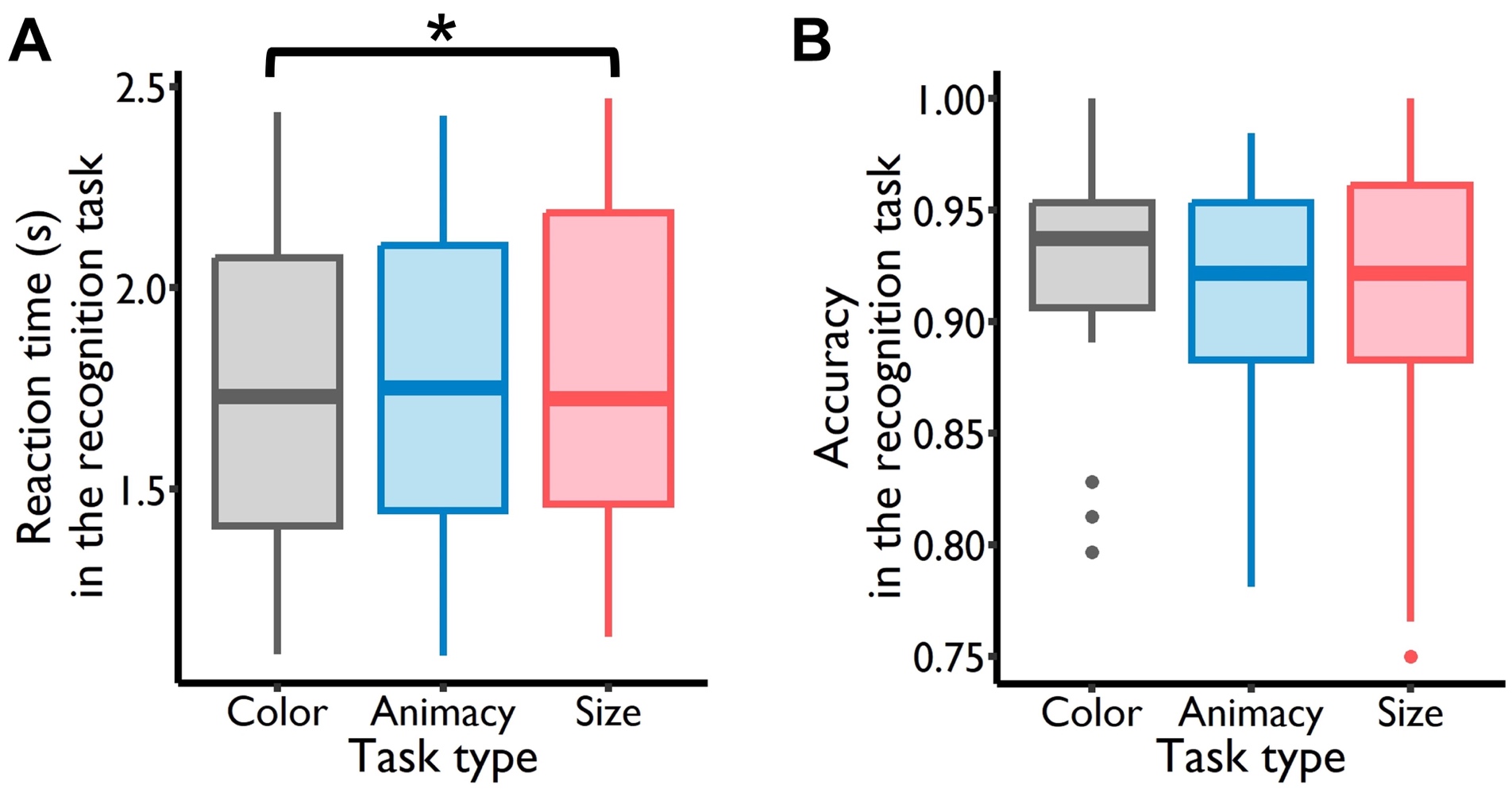


**Figure S1. The behavioral results in the recognition task.** (**A**) **Reaction time.** The mean reaction times of the object recognition under the color, animacy and size task conditions were 1.732 s (*SD* = 0.403 s), 1.766 s (*SD* = 0.402 s), and 1.822 s (*SD* = 0.412 s). The task type predicted the reaction times (*F_2, 52_* = 4.255, *p* = 0.019). Post-hoc testing showed that the reaction time of the object recognition under the color task condition was significantly faster than that under the size task condition (*t_52_* = -2.889, *p_corrected_* = 0.015), while the difference between the animacy task condition and the other two task conditions was not significant (animacy condition vs. color condition: *t_52_* = 1.097, *p_corrected_* = 0.520; animacy condition vs. size condition: *t_52_* = -1.793, *p_corrected_* = 0.182). (**B**) **Accuracy.** The mean accuracy of the object recognition under the color, animacy and size task conditions was 92.59% (*SD* = 5.44%), 91.38% (*SD* = 5.12%) and 91.55% (*SD* = 6.21%). The task type didn’t predict the accuracy of object recognition under three task conditions (*F_2, 52_* = 1.181, *p* = 0.315).

**Figure S2. Elekta Neuromag MEG channel positions.** Channels corresponding to different lobes are color-coded (figure adapted from www.megwiki.org)


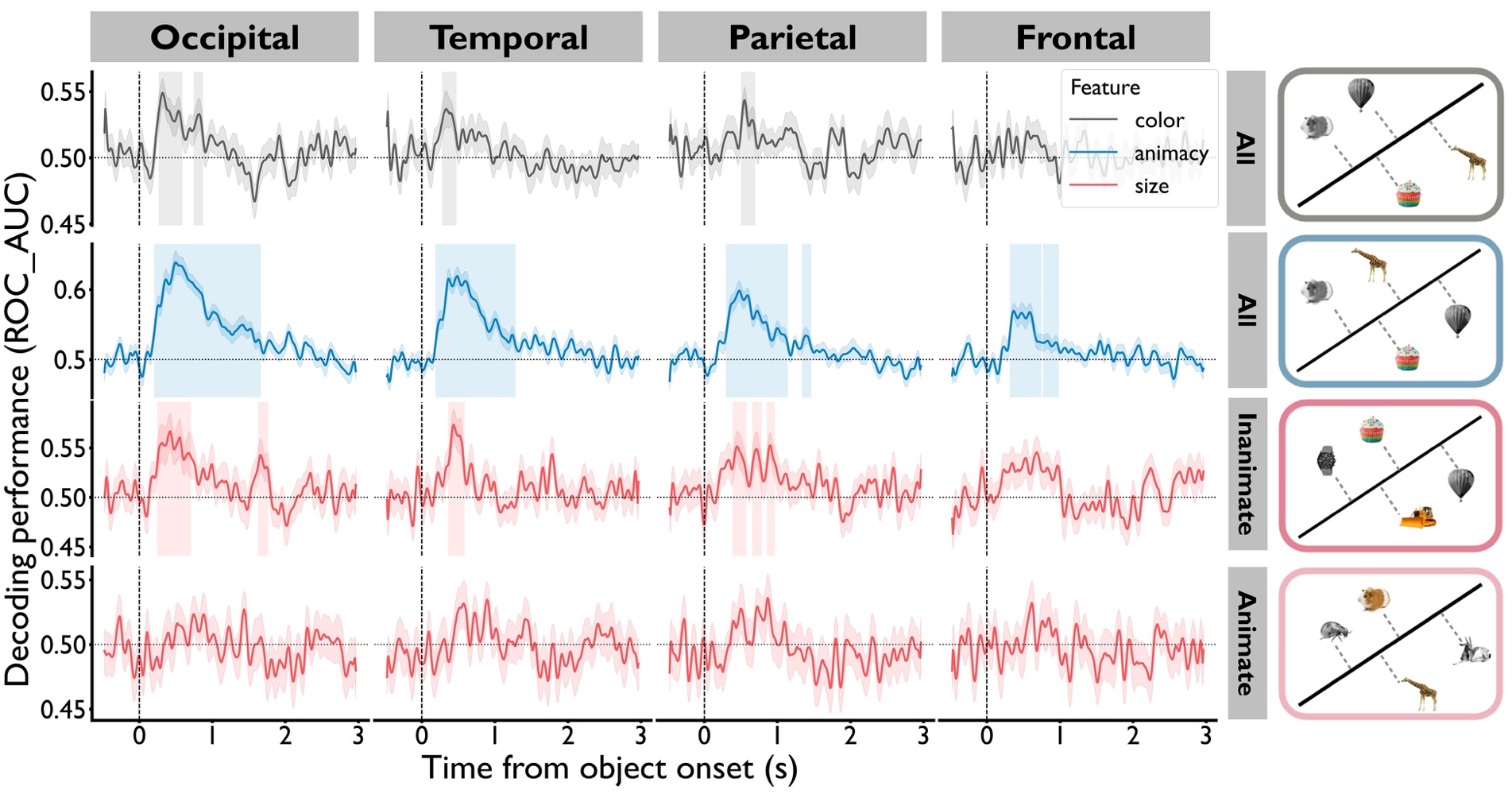


**Figure S3. The decoding performance of three features during the learning stage.** Significant clusters of color, animacy and size features in the occipital, temporal and parietal cortex were found (*P_corrected_* < 0.05). In the frontal cortex, only significant clusters of the animacy feature were found (*P_corrected_* < 0.05). There were no significant clusters of size feature for the animate objects. The shaded areas surrounding the classification performance time courses indicated standard error across participants. The vertical shaded areas marked the significant clusters carrying the feature information.


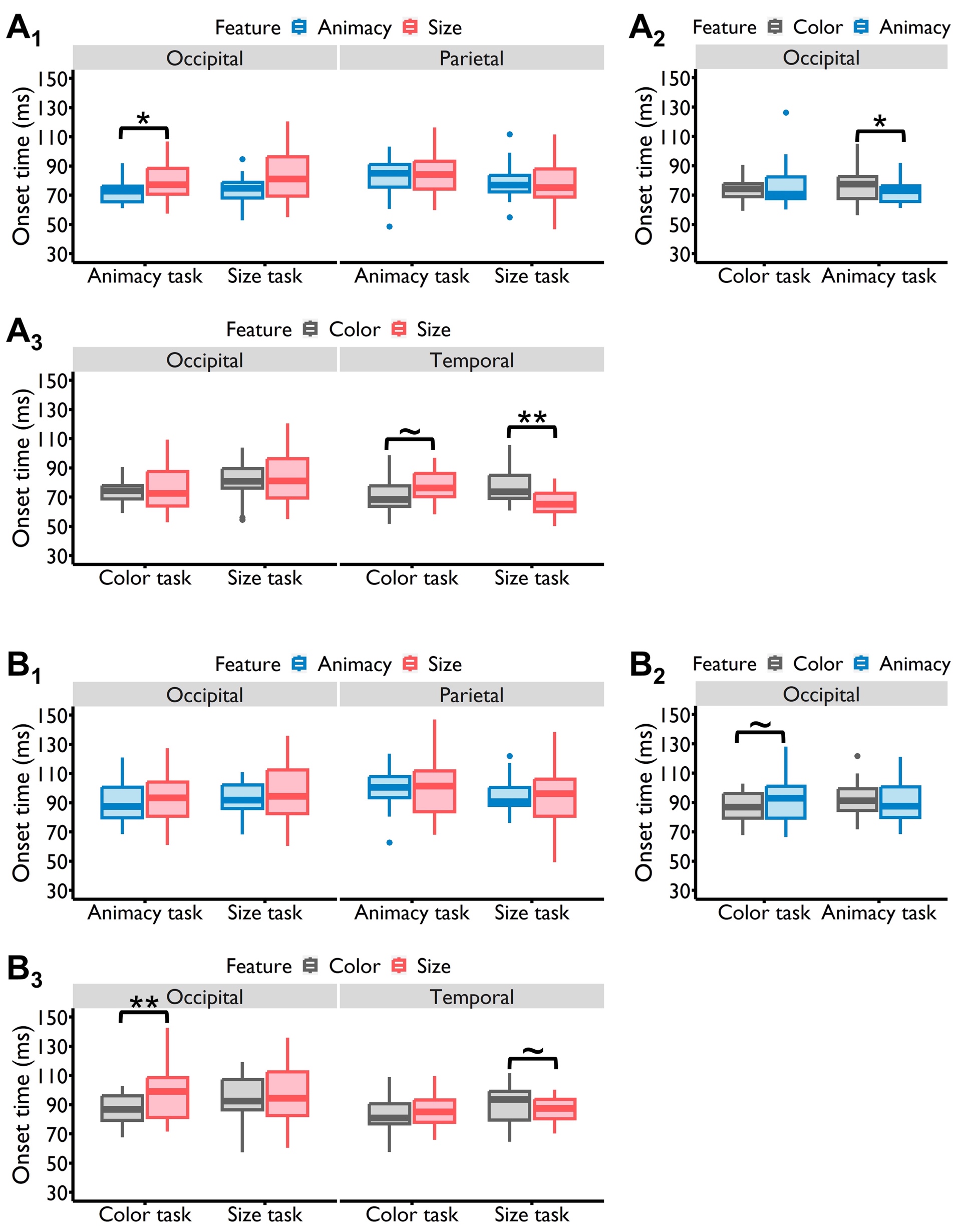


**Figure S4. The modulation effects of selective attention when setting T=30 ms (A) or 40 ms (B)**, i.e., the time window of at least 30 ms or 40 ms in which the d-values of all time points exceeding the corresponding threshold was considered as a significant cluster containing feature information. As shown in the figures, when the minimum duration (T) of a significant cluster increased, the overall onset times of features were delayed accordingly, and the patterns of time lags between different features remained discernible, although some of them became obscured.


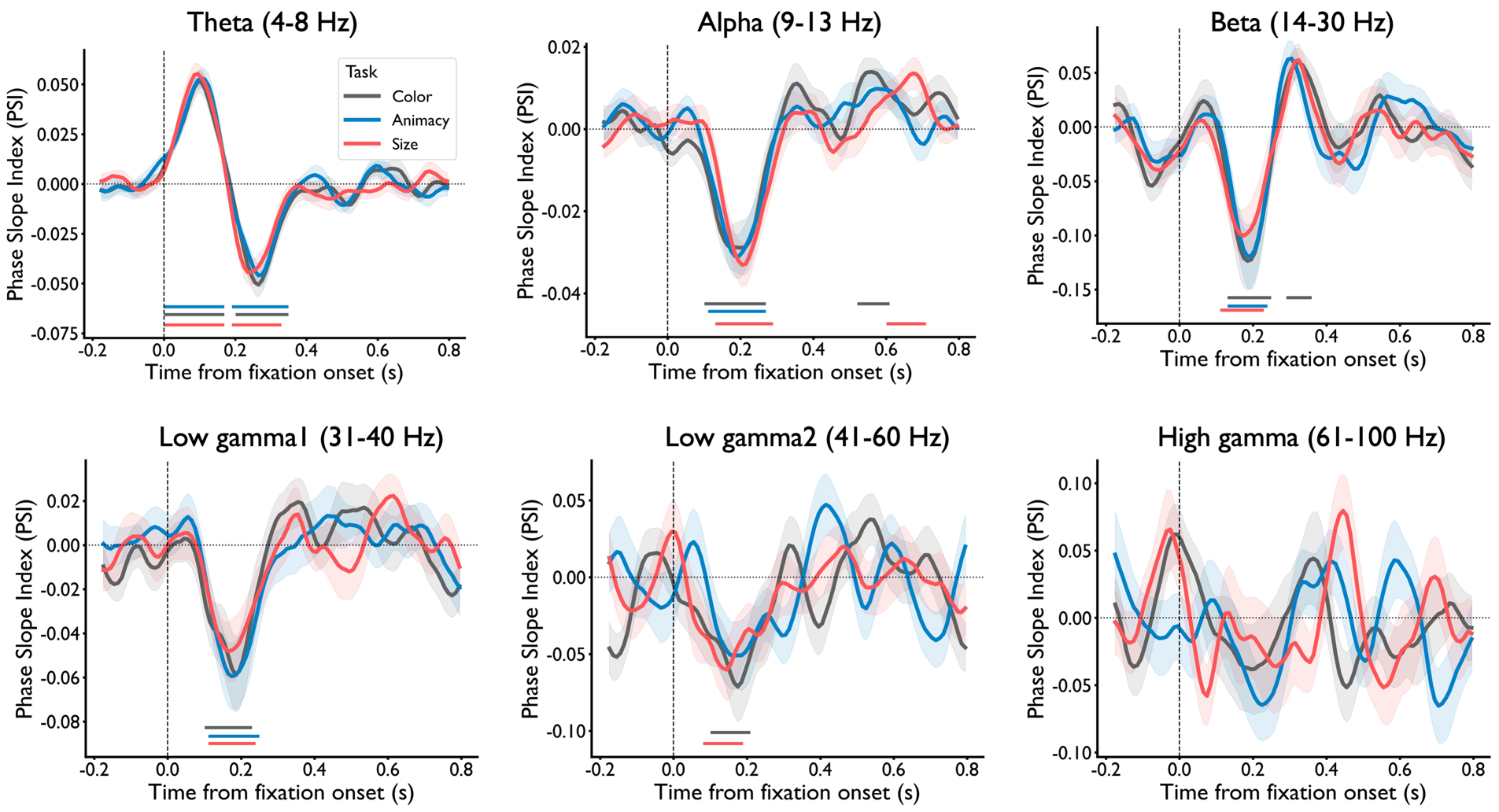


**Figure S5. Phase coupling patterns between the temporal and occipital lobes across different frequency bands.** Only phase coupling in the theta band demonstrated a pattern of initially top-down followed by bottom-up interactions. In contrast, the phase couplings in the alpha, beta, and low gamma bands were predominantly bottom-up, indicating an information flow from the occipital to temporal lobes. No significant phase coupling was observed in the high gamma band.


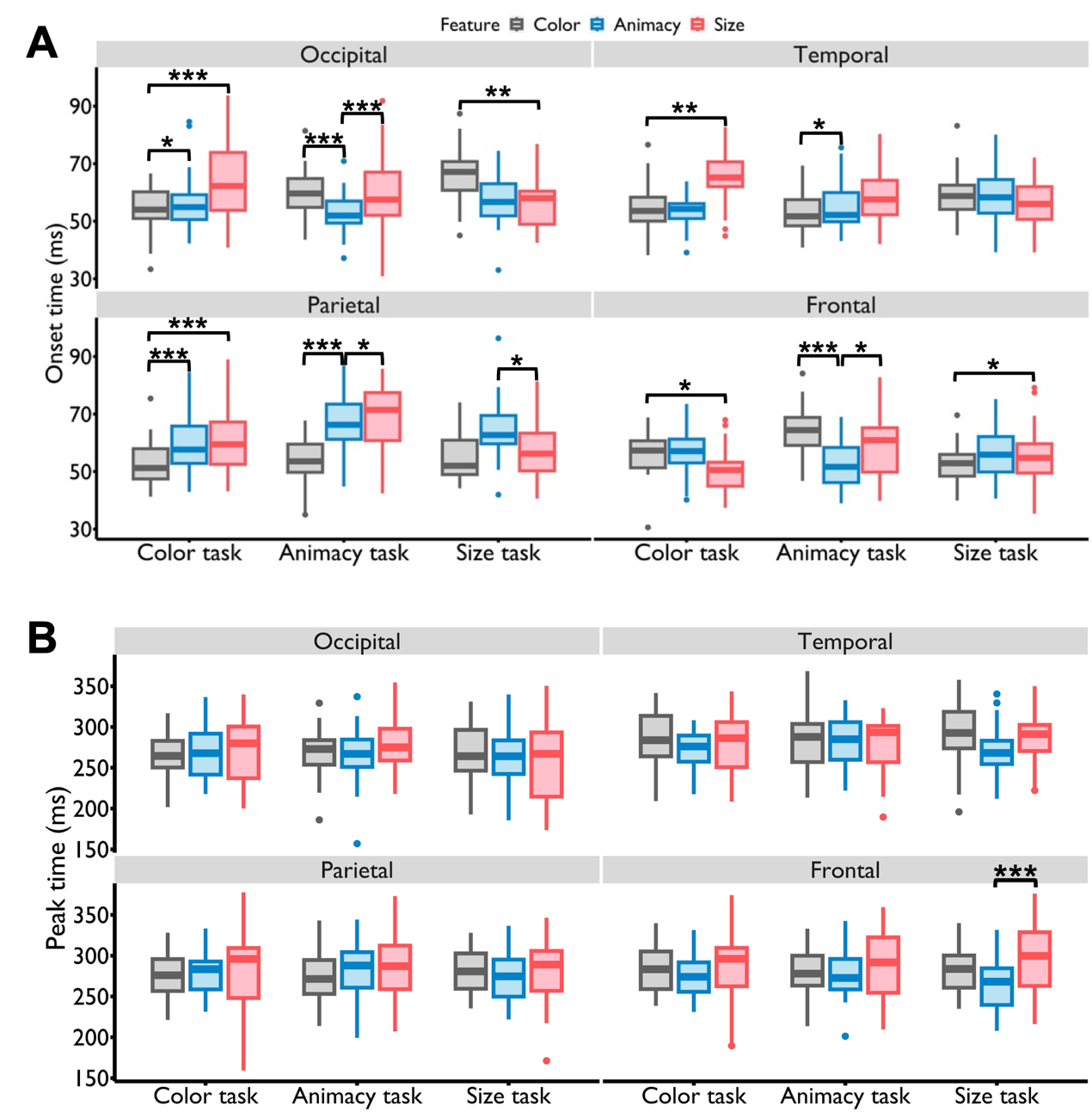


**Figure S6. Selective‑attention modulation of feature latencies during the perception task. (A) Onset latencies. Animacy vs. Size.** Occipital ROI: animacy onsets were earlier than size onsets in the Animacy task, whereas no reliable difference appeared in the Size task. Parietal ROI: the pattern reversed—animacy led in the Animacy task, but size led in the Size task. **Colour vs. Animacy.** Occipital ROI: colour onsets preceded animacy in the Colour task but were delayed relative to animacy in the Animacy task. **Colour vs. Size.** Occipital ROI: colour onsets preceded size in the Colour task yet lagged behind size in the Size task. Temporal ROI: colour led size in the Colour task, with no significant difference in the Size task. **(B)** **Peak latencies.** No significant attentional modulation was observed for peak latencies with any feature pair during visual perception. Significance: *: *p* < 0.05, **: *p* < 0.01, ***: *p* < 0.00 (FDR‑corrected). ROI = region of interest.


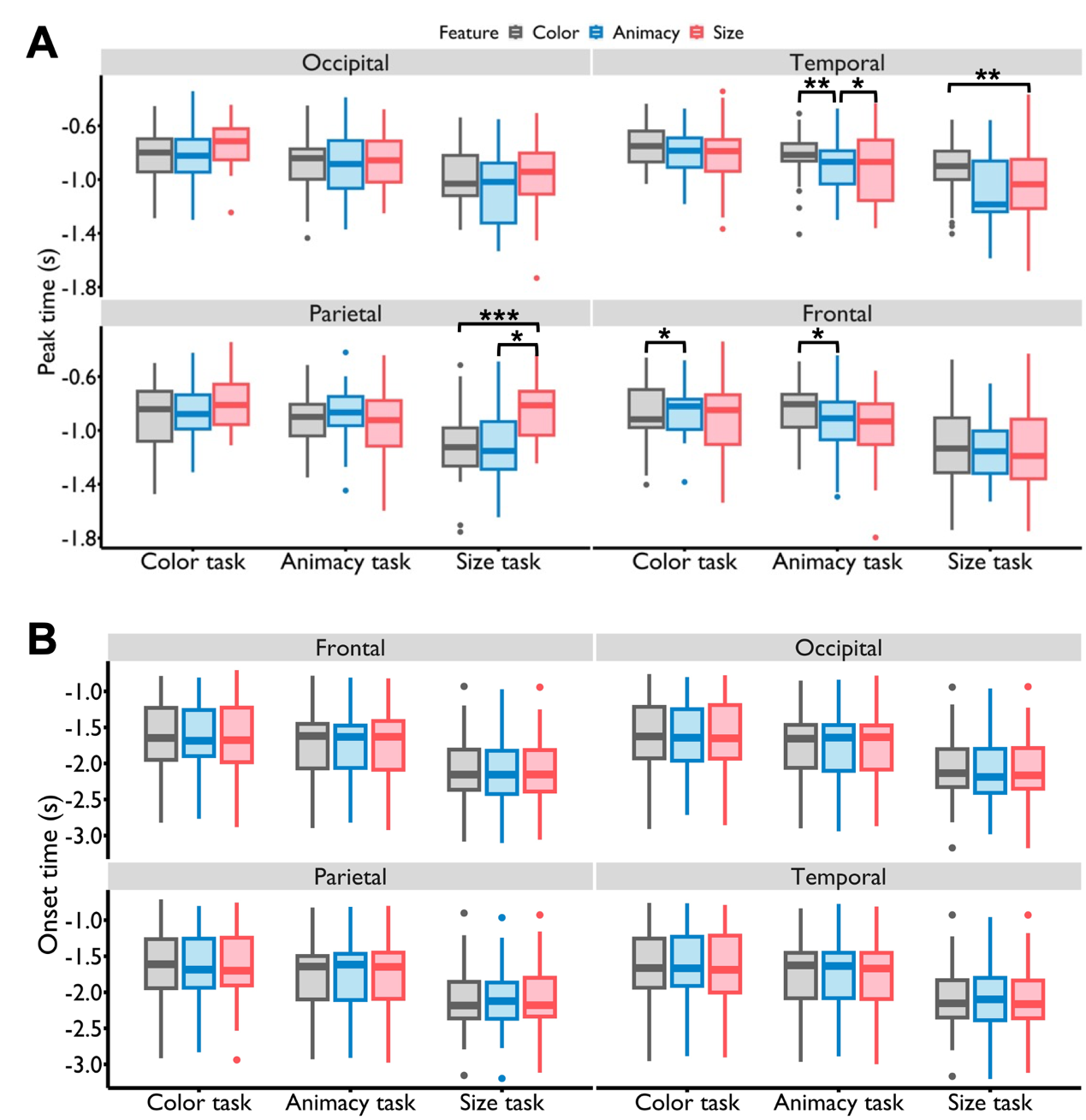


**Figure S7. Selective‑attention modulation of feature latencies during memory retrieval. (A) Peak latencies.** **Animacy vs. Size.** Temporal ROI: animacy peaks occurred earlier than size peaks in the Animacy task; no difference in the Size task. **Colour vs. Animacy.** Frontal ROI: a bidirectional effect—colour peaked earlier than animacy in the Colour task, whereas animacy peaked earlier than colour in the Animacy task. Temporal ROI: animacy peaked earlier than colour in the Animacy task; no difference in the Colour task. **Colour vs. Size.** Temporal ROI: size peaked earlier than colour in the Size task; no difference in the Colour task. **(B)** **Onset latencies.** No significant attentional modulation was detected for onset latencies with any feature pair during memory retrieval. Significance: *: *p* < 0.05, **: *p* < 0.01, ***: *p* < 0.00 (FDR‑corrected). ROI = region of interest.
